# Spatiotemporal coupling coordination between population aging and healthcare resource capacity in China: a provincial-level analysis, 2000–2023

**DOI:** 10.3389/fpubh.2026.1835598

**Published:** 2026-09-23

**Authors:** Qiang Liu, Guihua Xie

**Affiliations:** 1 College of Resources and Environmental Engineering, Tianshui Normal University, Tianshui, China; 2 Institute of Soil and Water Conservation, Chinese Academy of Sciences and Ministry of Water Resources, Yangling, China

**Keywords:** China, healthcare resources, population aging, regional disparity, spatiotemporal coupling coordination

## Abstract

**Introduction:**

China is experiencing rapid population aging with marked regional disparities, posing sustained challenges for healthcare provision. This study examines the spatiotemporal coupling coordination of two provincial composite indices, one summarizing population aging characteristics and one summarizing healthcare resource capacity, across 31 provincial-level regions, 2000–2023 (713 valid province-year observations).

**Methods:**

Aging was measured by five demographic and health indicators, capacity by six supply-side indicators covering hospitals and primary healthcare institutions; principal component analysis and the entropy method were applied in parallel and cross-validated (Pearson *r* = 0.94) before entering a coupling coordination degree model. The coupling coordination degree quantifies the statistical correspondence between the two composite indices. It does not measure whether the healthcare needs of older adults are met by services supplied, and supports no causal interpretation; because provincially consistent data specific to older adults (geriatric beds, long-term care facilities, geriatricians) were unavailable for 2000–2023, the index reflects general healthcare capacity rather than provision specific to older adults.

**Results:**

China’s aging coefficient rose from 7.0% (2000) to 14.9% (2023), following an acceleration–stabilization pattern with marked spatial heterogeneity. Hospital beds reached 9.10 million (≈6.4 per 1,000 population) and licensed physicians 4.28 million by 2023, up 77.6% since 2010, while the normalized healthcare composite rose from 0.02 to 0.91. National coordination improved across the period, moving from the lower to the upper bound of the pooled min–max normalization; these bounds denote the sample minimum and maximum rather than absent or perfect coordination. Regional disparities nevertheless persisted: Shandong and Jiangsu recorded values in the highest class of the classification adopted here (D ≥ 0.60), whereas Tibet and Qinghai remained in the lowest (D < 0.20), and the coefficient of variation of provincial aging coefficients declined only from 0.41 to 0.38.

**Discussion:**

The study contributes a 24-year, all-province series built on one fixed indicator framework spanning the reforms of 2003, 2009 and 2016, and a rule-based provincial typology, summarized as a Four-Dimensional Collaborative Development Framework, that identifies where demographic structure and healthcare capacity correspond least closely. The findings are an input to regional healthcare planning; whether the measures they suggest improve the care older adults receive lies beyond this design.

## 1 Introduction

China is experiencing rapid population aging, completing over a few decades a demographic transition that unfolded across a considerably longer period in the earlier-industrializing countries (1, 2). According to the Seventh National Population Census, China’s proportion of people aged 65 and above doubled from 7.0% in 2000, when the country first crossed the UN threshold for an aging society, to 14.9% in 2023. Projections indicate this figure will exceed 20% by 2035 and approach 30% by 2050, indicating a transition from an aging to a super-aged society that would run faster than the transitions recorded in most major economies (National Bureau of Statistics of China). With 264 million people aged 60 and above in 2023, China holds a large share of the world’s older population, which places sustained demands on its healthcare system (3).

The spatial heterogeneity of China’s aging process compounds these challenges (4). Eastern and northeastern regions such as Shanghai (18.7%) and Liaoning (20.0%) have already entered super-aged status, while western provinces such as Tibet (6.8%) and Xinjiang (9.2%) maintain relatively young populations, creating “multiple aging Chinas” within national borders. This pronounced heterogeneity intersects with the urban–rural divide, where rural areas experience accelerated aging due to youth out-migration, leaving behind older populations with limited healthcare access (5). Moreover, older populations use healthcare differently from younger ones. The prevalence of chronic conditions is higher, care is required continuously rather than episodically, and per capita expenditure is correspondingly greater (6, 7). We attach no specific national percentages to these statements, since figures defined consistently across the whole study period were not available to us.

China’s healthcare system faces structural inadequacies in addressing these complex care needs among older adults. Throughout this paper, healthcare resource capacity refers to the stock of healthcare institutions, beds, and health technicians recorded for a province in a given year. This is a supply-side quantity measure. It does not capture the administrative and financial mechanisms by which resources are assigned across regions, the efficiency with which those resources are used, or the geographic and financial accessibility of services to any particular population group, including older adults. Where the term allocation is used in this paper, it refers descriptively to the observed distribution of capacity across provinces and not to an allocation mechanism. Elsewhere the measured subsystem is referred to as healthcare resource capacity throughout. Healthcare resources expanded substantially between 2000 and 2023, with hospital beds reaching 9.10 million and licensed physicians 4.28 million by 2023, yet their distribution remains uneven. Provincial analyses report a pronounced east–west gradient in healthcare resource density, the higher densities clustering in the eastern provinces and the lower ones in the west, with the inequality substantially larger when resources are assessed against geographic area than against population (8). Eastern provinces therefore face high demand alongside relatively abundant resources, while western regions confront accelerating aging on a thinner resource base (9). The 48-fold difference between the highest and lowest provincial scores is a difference in normalized index values rather than in physical resource quantities, and we did not compile comparably constructed indices for other countries against which it might be judged.

The relationship between demographic transition and health system capacity can be stated as a three-link chain. The first link is that population aging alters healthcare need in both level and composition: in Andersen’s behavioral model, age structure operates as a predisposing characteristic while chronic disease burden operates as a need factor (10), and epidemiological transition theory specifies the accompanying shift from infectious to chronic and degenerative burdens, which raises the demand for continuous rather than episodic care (2). Health systems then respond to changed need by adjusting the stock of institutions, beds, and health technicians. This adjustment is slow, capital-intensive, and administratively bounded: facilities once built and staff once trained persist, and provincial fiscal capacity constrains how quickly the stock can change, so adjustment exhibits path dependence and increasing returns (11–13). Because the demographic side changes continuously while the capacity side changes discretely and with a lag, the two systems do not converge to a fixed relationship; they co-evolve, and the state of their correspondence at any moment is itself the object of interest. This is the reason for adopting a coupling coordination perspective. A simple ratio, for example beds per person aged 65 and above, records the relative size of the two quantities but is insensitive to the developmental level of either system, so that a province with a small older population and a very small healthcare stock can score identically to a province where both are large. A regression framework, by contrast, requires a directional causal claim and an identification strategy that provincial aggregate data over 24 years cannot supply. The coupling coordination degree model occupies the intermediate position that the question requires: it summarizes the degree of correspondence between two co-evolving systems while conditioning that summary on the developmental level of both, and it does so without asserting a direction of causation.

The question addressed by this study is how closely two provincial composite indices have corresponded to one another across China’s provinces and over time, one of them summarizing the demographic structure of ageing and the other the stock of healthcare resources. The coupling coordination degree serves as a summary statistic of that correspondence between two co-evolving systems. It is not a measure of healthcare demand, of unmet need, of geographic or financial accessibility, or of the adequacy or quality of the care that older adults receive, and no such interpretation is intended in what follows. Provincial ageing trajectories and healthcare endowments differ substantially while national policy has been broadly uniform in design (14). Whether that uniformity, or the tendency of market allocation to favor economically developed areas, accounts for the patterns documented here is not tested by the present design, and it is raised only as context in Section 5.2.

This study examines the spatiotemporal coupling coordination between population aging and healthcare resource capacity across China’s 31 provincial-level regions from 2000 to 2023 (15). Four tasks follow from that aim. We document how population aging and the distribution of healthcare resources evolved in time and across space, noting where the trajectories turn or diverge. Composite indices are then constructed on several dimensions of aging characteristics and of healthcare capacity, which allows the correspondence between the two to be assessed on something other than a simple ratio. The coupling coordination degree is computed for every province and year from those two indices. Spatial clusters and regional patterns are described through mapping and classification, and from the joint distribution of the two indices we derive a rule-based provincial typology, setting out the planning priorities that follow from it without claiming that the instruments discussed have been evaluated.

Against the gap identified above, namely that existing work tracks aging and healthcare resources over shorter windows, within single regions or single cities, or through a single analytical method, and does not follow the joint evolution of aging and general healthcare capacity across all provincial-level units over the full period of China’s healthcare reforms, we state the contribution of this study explicitly. The principal contributions of this study are threefold. (i) Coverage. We provide a longitudinal analysis spanning 24 years and all 31 provincial-level units, whereas coupling coordination analyses of aging and healthcare resources in China have been confined to shorter windows or to single basins (16, 17). The period covered encompasses the demographic and healthcare system transitions associated with major reforms, including the New Rural Cooperative Medical Scheme (2003), the New Healthcare Reform (2009), and the Healthy China 2030 strategy (2016). (ii) Measurement. We claim no methodological novelty for principal component analysis, the entropy weight method, or the coupling coordination degree model; all three are well established and are used here as standard instruments. The contribution lies in the construction and consistent application of a two-system indicator framework that is held fixed across 31 provinces and 24 years, in the parallel computation and cross-validation of two independent weighting schemes (Pearson *r* = 0.94), and in the weighting and imputation sensitivity analyses reported in Sections 3.3.4 and 3.4. Together these make the coordination series comparable across provinces and over time, which is the precondition for the spatiotemporal comparison that constitutes the substance of the study. (iii) Typology. We characterize the coexistence of several aging regimes within one country, which we term “multiple aging Chinas”, and derive from the joint distribution of aging and resources a Four-Dimensional Collaborative Development Framework that sets out regionally differentiated strategies for the healthcare of older adults across China’s territory. Taken together, these contributions help to address gaps in understanding the dynamic relationship between demographic transition and health system capacity, and provide analytical tools that may inform policy formulation in aging societies facing spatial inequalities.

## 2 Literature review

### 2.1 Theoretical framework

This study draws on three bodies of theory, introduced here before the Methods so that the analysis can be read against them. The first is health service demand theory. Following Andersen’s Behavioral Model of Health Services Use (10), population aging shapes healthcare demand through predisposing characteristics (age structure), enabling resources (accessibility), and need factors (chronic disease burden), so that growth in the older population alters both the level and the structure of demand. The second is regional development coordination theory. Coupling coordination theory, which originates in systems science, treats two subsystems as interacting and co-evolving, and provides analytical tools for examining the correspondence between demographic structure and resource allocation. The third is health equity theory. Following the definition of equity as the absence of systematic differences in health between groups with differing social advantage (18), and the policy framework for healthy ageing set out in the WHO World report on ageing and health (1), resource allocation is judged against population need, with horizontal equity (equal resources for equal need) and vertical equity (differentiated resources for differing need).

Three further strands inform the interpretation of the results. Epidemiological transition theory describes the staged shift from infectious to chronic and degenerative disease burdens that accompanies demographic transition, and supplies the expectation of gradual progression against which China’s compressed and spatially uneven transition is read (2). The inverse care law holds that the availability of good medical care tends to vary inversely with the need of the population served, and that this operates more completely where care is exposed to market forces (19). Institutional accounts of humanly devised constraints (11) and of increasing returns and path dependence (12), together with institutional complementarity (20) and agglomeration under increasing returns in economic geography (13), help to explain why allocation patterns, once established, tend to persist. Local indicators of spatial association (21) provide the method by which such persistence could be tested formally.

### 2.2 Empirical studies on population aging and healthcare resource allocation

A first strand documents the consequences of population aging for health systems and the demand side of care. Conceptual and country-level work sets out how aging reshapes health-system requirements and links the health of older adults to healthcare resources and fiscal outcomes (6, 22). Reviews and case studies extend this to lower- and middle-income and developing-country settings, where access, affordability and service models are recurring concerns (14, 23–27). At the individual level, utilization among older populations has been shown to vary with multidimensional health status (7), and technology-oriented and workforce responses, including digital and platform-based management and changes in skill-mix, have been proposed to meet rising and changing demand (28–31).

A second strand concerns the criteria and methods for allocating healthcare resources. Reviews of allocation approaches set out the principles against which distributions are assessed (5), including debates on age rationing (32). Empirical work in China has ranked the resource factors most associated with public satisfaction (33) and examined how allocation interacts with patient choice in rural areas (34). At national scale, provincial healthcare resources have been shown to cluster spatially and to be distributed far less equally against geographic area than against population over 2010 to 2021 (8), while the relation between the level of economic development and that distribution has been examined for 2001 to 2020 (9). Inequality in human resources for health has been linked to mortality across 172 countries and territories (35), which places the subnational Chinese pattern within a broader distributional literature.

A third strand addresses accessibility and the spatial dimension of supply. Accessibility measures have been refined to incorporate the temporal dimension and age-specific demand (15), and place has been shown to shape how people navigate care (36). Sub-national analyses have documented hierarchical accessibility within a Chinese city (37) and spatial mismatch between primary care and older populations in South Korea (38). The same spatial logic has been applied prospectively rather than diagnostically, in decision-support approaches to siting new facilities for aging populations (39).

A fourth strand applies matching and coupling coordination models directly to aging and healthcare resources in China. Work in this vein covers 2008 to 2017 with a matching index (40), 2011 to 2020 with spatial autocorrelation and a Bayesian spatiotemporal specification (41), 2012 to 2022 with the coupling coordination degree at national scale (16), 2013 to 2020 for thirty provinces alongside a productivity subsystem (42), a single basin (17), and urban older-adult care facilities rather than general healthcare capacity (43). Together these establish that the coupling coordination degree model transfers workably from its origins in economy-environment analysis to the aging-health domain, and that equal weighting of the two subsystems is the established convention in that transfer. What they do not establish is how coordination behaves over a long horizon across the full set of subnational units. The national-scale application (16) cannot exhibit interprovincial divergence because the provinces are aggregated away, while the single-basin application (17) observes divergence only among the provinces of one basin, so that the eastern-western contrast dominating the national picture falls outside its frame. None of these windows spans the sequence of reforms from 2003 to 2016, so none can indicate whether coordination improved through reform or independently of it.

### 2.3 Points of convergence and divergence

Across these strands there is broad agreement on several points. Population aging raises the level of healthcare demand and shifts its composition toward chronic and long-term care (6, 7). The distribution of resources is spatially uneven and place-dependent, so that geographic context conditions access (36–38). Where direct measurement of demand is difficult, supply-side indicators are widely accepted as proxies for system capacity (5, 15).

The strands diverge in scale, method and focus. Coupling coordination applications are set at the national level (16) or within a single basin (17); accessibility studies are set within a single city (37) or in another national context (38); and allocation and satisfaction studies are largely cross-sectional (5, 33, 34). They also differ in emphasis, with some centered on the demand side (7, 22), others on supply and its criteria (5, 32, 33), and others on the alignment of the two (16, 17). A point of disagreement concerns the role of markets: the inverse care law anticipates that market allocation widens the gap between provision and need (19), whereas need-based and equity-oriented accounts hold that allocation can be steered toward need (1, 18). These positions have not been adjudicated against long-run subnational evidence.

### 2.4 Research gap

Taken together, the literature establishes that aging reshapes demand, that supply is unevenly distributed, and that coupling coordination is a workable way to assess the correspondence between the two. What remains unexplored is the joint evolution of aging and general healthcare capacity across all 31 provincial-level units over the full period of China’s healthcare reforms. Existing analyses of the correspondence between ageing and healthcare resources in China are set within shorter windows, within single regions, or within a single urban system (16, 17, 40–43); accessibility analyses are confined to a single city (37) or to settings outside China (38); and allocation-criteria and satisfaction studies are not spatiotemporal (5, 32, 33). None of those windows spans the reforms of 2003, 2009 and 2016, and none holds a single indicator framework fixed across all 31 provincial-level units for the full period, which is the precondition for comparing coordination across provinces and over time rather than within one window. The two literatures have largely developed apart. Studies of population aging characterize the demographic process and its implications for need but treat health system capacity as background; studies of healthcare resources characterize the distribution of capacity and its determinants but treat the age structure as a control variable. Where the two have been brought together through coupling coordination models, the analysis has been confined to a single scale, a single region, or a short window (16, 17). The interaction between a compressed demographic transition and a reforming health system, observed across all provincial-level units and across the 24 years spanned by China’s major healthcare reforms, therefore remains unexamined, and it is this gap that the present study addresses.

## 3 Materials and methods

### 3.1 Study design and data sources

This study employs a longitudinal panel design analyzing provincial-level data spanning from 2000 to 2023 across 31 Chinese provincial administrative regions. The study area encompasses mainland China’s diverse geographic and socioeconomic landscape, covering approximately 9.6 million square kilometers and representing a rapidly aging society. China’s vast territory exhibits considerable heterogeneity in both natural conditions and socioeconomic development, making it a suitable setting for examining spatial variations in aging-healthcare resource relationships. The eastern coastal regions, characterized by developed economies and high population densities, contrast sharply with the western interior provinces featuring mountainous terrain, lower population densities, and developing economies. This geographic diversity is further complicated by the country’s distinctive administrative structure, comprising 22 provinces, 5 autonomous regions (Inner Mongolia, Guangxi, Tibet, Ningxia, and Xinjiang), and 4 directly administered municipalities (Beijing, Tianjin, Shanghai, and Chongqing). Each administrative unit possesses distinct demographic characteristics, economic development levels, and healthcare infrastructure, contributing to the complex spatial patterns of population aging and healthcare resource distribution. Hong Kong Special Administrative Region, Macau Special Administrative Region, and Taiwan Province were excluded from this analysis due to data unavailability and differences in healthcare system structures (Figure 1).

**Figure 1:**
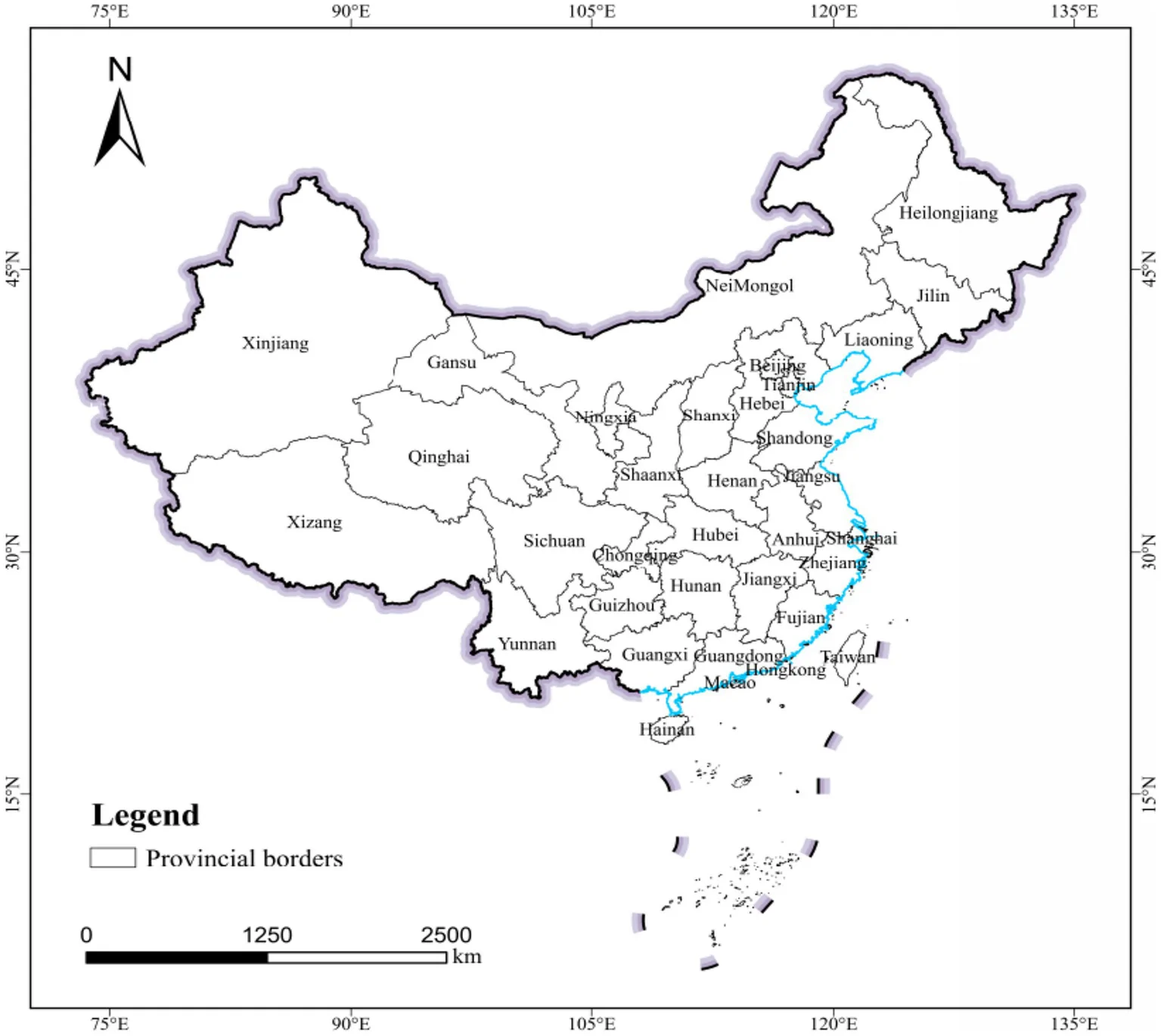
The study area of China.

The selection of the 2000–2023 study period covers a phase of marked demographic transition in China, encompassing the implementation of major healthcare reforms, rapid economic growth, and accelerating population aging. This 24-year timeframe allows for robust analysis of long-term trends while maintaining data consistency and comparability. During this period, China experienced substantial socioeconomic transformations, including massive rural-to-urban migration, implementation of the New Rural Cooperative Medical Scheme (2003), urban health insurance reforms, and the gradual relaxation of the one-child policy, which influenced both population aging patterns and healthcare resource allocation (34).

Primary data on older populations and healthcare resources were systematically collected from the China Health Statistical Yearbook series (2000–2023), published annually by the National Health Commission. The theoretical number of observations in this study was 744, with 713 valid observations actually obtained and 31 missing. Missing data primarily originated from Tibet and Xinjiang, where early health statistical systems were incomplete, rendering data for certain years unavailable from official statistical yearbooks.

For the aforementioned missing data, this study employed linear interpolation for imputation. Specifically, for missing values of a particular variable in a specific province and year, we estimated the values using linear interpolation based on data from the nearest available years before and after the missing period. Linear interpolation was chosen for several reasons. The healthcare resource and population indicators in this study exhibit smooth and gradual temporal trends, satisfying the basic assumptions of linear interpolation; missing data were concentrated at the beginning of the time series (2000–2003) with a low missing proportion (4.2%), and linear interpolation can utilize information from adjacent years for reasonable estimation; and linear interpolation is widely adopted in panel data analysis and represents one of the standard methods for handling time-series missing data. The missing observations fall in 2000–2003, a period critical to the onset of healthcare reform and population aging in China, and interpolation may attenuate genuine short-run fluctuations in these early years; this is acknowledged as a limitation of the present study. The affected observations are confined to Tibet and Xinjiang and represent 4.2% of all province-year observations, that is 31 of 744. They are low-resource western province-years whose values lie close to the floor of the distribution and therefore carry limited weight in the national aggregates and in the two-phase acceleration–stabilization pattern reported below.

This authoritative source provides comprehensive, standardized data on demographic characteristics, health status indicators, and healthcare infrastructure across all provincial units. The yearbook’s consistent methodology and quality control procedures support data reliability and temporal comparability. Supplementary demographic data were obtained from national population censuses conducted in 2000, 2010, and 2020, with intercensal years estimated using official annual population surveys. Land area data for calculating population density indicators were sourced from the National Bureau of Statistics’ most recent territorial survey update in 2018, which accounts for administrative boundary adjustments and provides measurements for each provincial unit.

### 3.2 Variable construction

The construction of variables for this study followed a systematic approach designed to capture the multifaceted nature of population aging and healthcare resource availability. The selection of indicators was guided by three complementary approaches. We began with correlation analysis among potential indicators, retaining those with correlation coefficients below 0.8 to limit multicollinearity while retaining comprehensive coverage. The indicators were then aligned with international standards established by WHO’s Framework for Countries to Achieve an Integrated Continuum of Long-term Care (2021) and UNFPA’s Ageing in the Twenty-First Century framework, supporting comparability with global studies. Selection was finally checked against a literature review of 47 peer-reviewed studies on aging-healthcare dynamics in China (2015–2023), indicating that our chosen indicators are among the more frequently utilized measures (appearing in >70% of reviewed studies).

For the assessment of population aging, we developed five core indicators that comprehensively reflect demographic and health dimensions. The population aged 65 years and above, measured in tens of thousands of persons, serves as the fundamental quantitative indicator of the size of the older population in each province. This absolute measure provides the baseline for understanding the scale of aging across different regions. To account for geographic variations in provincial sizes, we calculated the density of the older population as the number of persons aged 65 and above per square kilometer, enabling meaningful comparisons between geographically diverse regions such as densely populated eastern provinces and vast western territories.

The aging rate, calculated as the percentage of the population aged 65 and above relative to the total population, is a widely used international indicator for comparing population aging levels across different societies. This proportional measure allows for standardized comparisons unaffected by absolute population sizes. Complementing this, the old-age dependency ratio was computed as the ratio of the population aged 65 and above to the working-age population (15–64 years), multiplied by 100. This indicator captures the potential economic burden of population aging on the productive population and reflects the social support pressure in each region. Life expectancy at birth, measured in years, serves as a comprehensive proxy for overall population health status and healthcare system effectiveness, as longer life expectancy typically indicates better health conditions and healthcare access throughout the life course.

For healthcare resource assessment, we constructed six indicators spanning two major categories of China’s healthcare delivery system. Within the hospital category, we measured the total number of hospitals in each province, encompassing general hospitals, traditional Chinese medicine hospitals, integrated Chinese-Western medicine hospitals, ethnic medicine hospitals, various specialized hospitals, and nursing homes. Hospital bed capacity was measured as the actual number of beds available in tens of thousands, representing the physical infrastructure for inpatient care. Hospital health technical personnel, also measured in tens of thousands, includes all licensed physicians, registered nurses, pharmacists, laboratory technicians, and other professional staff.

The primary healthcare institution indicators parallel the hospital measures but focus on grassroots facilities that provide first-contact care and ongoing management for older adults. The number of primary healthcare institutions encompasses community health service centers and stations in urban areas, township and street health centers in suburban areas, village clinics in rural areas, and various outpatient departments and clinics. Primary healthcare bed capacity and health technical personnel were measured using the same units as hospital indicators, capturing the infrastructure and human resources available for basic healthcare delivery at the community level (6).

The use of a comprehensive index to characterize population aging characteristics, rather than single demographic measures, reflects the multidimensional nature of population aging and its healthcare implications. Unlike simple age proportions, our comprehensive approach captures the complex interplay between demographic structure, spatial distribution, and health status that collectively determine healthcare demand patterns.

Regarding healthcare resources for older adults, we acknowledge that standard hospital and primary care indicators may not fully capture specialized geriatric care needs. To address this limitation while maintaining data consistency across the 24-year period, we incorporated proxy measures within our framework: life expectancy serves as an outcome indicator reflecting healthcare system effectiveness for older adults, while the density of primary care facilities captures accessibility for older adults with limited mobility (Table 1).

**Table 1:** Research index system for population aging and healthcare resources.

System	Dimension	Indicator	Theoretical basis	Rationale for selection
Population aging	Population quantity	Population aged ≥65 years (10,000 persons)	Andersen predisposing characteristics (10)	Absolute size of the population that generates the largest share of utilization; required so that the index is not driven by rate alone.
	Population density	Density of population aged ≥65 (persons/km^2^)	Economic geography; agglomeration (13)	Distinguishes provinces with the same share of older adults but very different settlement patterns, which bear on service delivery.
	Population structure	Aging rate (%)	Demographic transition; international comparability	Standard internationally comparable measure of the depth of aging; anchors the six-stage classification.
		Old-age dependency ratio (%)	Andersen predisposing characteristics (10)	Captures the burden borne by the working-age population; entered as a reverse indicator (see Section 3.3.2).
	Health status	Life expectancy at birth (years)	Epidemiological transition theory (2)	Proxy for the stage of the health transition and for the duration of the period of elevated need.
Healthcare resource capacity	Hospitals	Number of hospitals	Andersen enabling resources (10)	Institutional density of the higher tier of the delivery system.
		Hospital beds (10,000)	Andersen enabling resources (10)	Standard capacity measure for inpatient and chronic care, the tier used most heavily by older adults.
		Hospital health technicians (10,000 persons)	Health workforce; skill-mix literature (29, 31)	Human resource capacity; buildings without staff do not constitute capacity.
	Primary healthcare	Number of primary healthcare institutions	Health equity theory; horizontal equity (1, 18)	Primary tier is the point of first contact and the main site of chronic disease management for older adults.
		Primary healthcare beds (10,000)	Inverse care law (19)	Distinguishes provinces whose capacity is concentrated in the hospital tier from those with a developed primary tier.
		Primary healthcare technicians (10,000 persons)	Health equity theory; vertical equity (1, 18)	Staffing of the tier most relevant to geographic reach outside provincial capitals.

We acknowledge that the healthcare resource indicators employed in this study are comprehensive indicators designed for the general population rather than indicators specific to older adults. Ideally, the study should incorporate specialized indicators such as the number of geriatric beds, rehabilitation and nursing facility counts, long-term care facility capacity, and the number of geriatric specialists. However, systematic statistics on healthcare resources specific to older adults at the provincial level in China remain incomplete for the 2000–2023 period, as existing yearbooks and official statistical sources do not provide temporally consistent specialized data. General healthcare resources therefore bound, but do not equal, the capacity to serve older adults: the indicators used here measure the general capacity of the provincial healthcare system on which the older population draws, and they do not capture geriatric beds, long-term care and rehabilitation facilities, or geriatricians. The coupling coordination degree is accordingly a first-order structural indicator of the alignment between general healthcare capacity and demographically driven demand pressure, and no claim is made about the adequacy of services specific to older adults. The incorporation of specialized geriatric indicators, as China’s statistics on care for older adults mature, remains a priority.

Despite these data limitations, comprehensive healthcare resource indicators can serve as valid proxies for the capacity to serve older adults, for several reasons. Older adults are the primary users of healthcare resources, those aged 65 and above in China consume over 45% of healthcare resources, with higher outpatient visit frequencies and hospitalization rates than other age groups, so that comprehensive healthcare resource allocation is closely related to the capacity to serve older adults. Hospitals and primary healthcare institutions are also the main venues where older adults access chronic disease management, inpatient treatment, and basic medical services, and their resource allocation levels are closely related to the accessibility of health services for older adults. In international research, moreover, adopting comprehensive healthcare resource indicators as proxy variables is a widely accepted research strategy when specialized data are unavailable, and numerous studies have employed similar approaches to analyze the relationship between aging and healthcare resources.

#### 3.2.1 Data processing

- *Directionality and standardization*: Of the eleven indicators, ten are positive and one, the old-age dependency ratio, is negative in the sense that higher values denote greater burden; it was reverse-transformed before standardization by the procedure set out in Section 3.3.2. All indicators were then rescaled to the unit interval by min−max standardization, as described in the same section. The minimum and maximum used in that standardization are the pooled minimum and maximum over all 713 province-year observations, not the annual extrema.
- *Missing data*: Of 744 theoretical province-year cells, 713 were observed and 31 (4.2%) were missing, all of them in Tibet and Xinjiang for 2000–2003, where provincial health statistical reporting was not yet complete. Missing cells were completed by linear interpolation between the nearest observed years. Four robustness checks were performed and are reported in Supplementary Tables S1–S4: re-derivation of all series with Tibet and Xinjiang excluded, which removes every imputed value; re-imputation under three alternative schemes (nearest observed value, cubic spline, province-specific linear extrapolation), under which the coordination series is almost unchanged (Pearson *r* = 0.972–0.981, Spearman ρ = 0.965–0.974, maximum |ΔD| ≤ 0.016); bounding, in which each imputed value is replaced by the lower and then the upper of its two nearest observed values, under which neither the aging-stage nor the coordination-level classification of the two provinces changes; and quantification of the effect on national composites, which shift by at most ±0.006 for 2000–2003.
- *Comparability across years*: Two features of the procedure are what make annual values comparable, and both are consequences of pooling rather than of annual recomputation. The entropy weights were computed once on the pooled panel of 713 observations and then applied unchanged to every province-year, so that a change in a province’s score between two years reflects a change in its indicator values and not a change in the weights. The min–max bounds applied in that standardization are likewise pooled, so the 0–1 scale is fixed across the whole period. Had weights or bounds been recomputed annually, each year would have carried its own scale and the temporal series would not have been interpretable. It follows that the values 0.00 and 1.00 are the pooled sample minimum and maximum and are not absolute states. We further note a residual source of non-comparability that standardization cannot remove: the statistical definition of primary healthcare institutions in the China Health Statistical Yearbook was adjusted during the study period, which affects the level of that indicator series; because the entropy weights and the coordination degree depend on relative rather than absolute magnitudes, and because the adjustment applies uniformly across provinces within a year, its effect on interprovincial comparison is limited, but it constrains the interpretation of the absolute level of the primary healthcare series over time.
- *Software and reproducibility*: Principal component analysis and the entropy weight computation were performed in SPSS 21.0; the coupling coordination degree, the sensitivity analyses and the imputation checks were computed in Python 3.9; mapping and natural-breaks classification were performed in ArcGIS 10.8. Every indicator series used here is drawn from published statistical sources, which are identified by title and year in Section 3.1 and listed by indicator in Table 1, so that the panel can be reassembled from the same publications without reference to our own working files. The compiled province-year panel that underlies the results, together with the parameter settings behind the composite indices, the coupling coordination degree and the sensitivity analyses of Sections 3.3.4 and 3.4, is available from the corresponding author on request. We are glad to place it in a public repository at the point of acceptance if the journal prefers that route.

### 3.3 Analytical methods

Our analytical framework employs a hierarchical, four-stage approach designed to systematically evaluate the spatiotemporal dynamics of population aging and healthcare resource allocation. The methodological sequence runs as follows. We calculate the aging coefficient as the basic demographic measure, then apply the entropy weight method at the national level to objectively assign weights to indicators based on their information content. After that, principal component analysis (PCA) is employed at the provincial level for dimensionality reduction while preserving maximum variance in the multidimensional data. Finally, the coupling coordination degree model synthesizes the outputs from previous stages to quantify the coupling coordination degree between population aging characteristics and healthcare resource availability.

#### 3.3.1 Aging coefficient

The aging coefficient serves as our fundamental measure for quantifying population aging intensity across China’s provinces. We define this coefficient as the proportion of the population aged 65 years and above relative to the total population, expressed as a percentage (16). The calculation of the aging coefficient for province *i* in year *t* follows Equation 1:


$${\text{Aging Coefficient}}_{\mathrm{it}}=\frac{\text{Population aged}\geq 65_{\mathrm{it}}}{{\text{Total Population}}_{\mathrm{it}}}\times 100$$ (1)

To enhance interpretability, we categorized provinces into six aging stages based on established demographic thresholds: young society (<4%), adult society (4–7%), lightly aging society (7–10%), moderately aging society (10–14%), highly aging society (14–16%), and aged society (>16%). These categories facilitate temporal tracking of demographic transitions and enable identification of provinces at similar aging stages for comparative policy analysis.

#### 3.3.2 Entropy weight method

To objectively synthesize multiple indicators into comprehensive evaluation scores at the national level, we implemented the entropy weight method. This information theory-based approach derives indicator weights from the data’s inherent variation, eliminating subjective bias inherent in expert-assigned weighting schemes.

Prior to applying the entropy weight method, we addressed the directionality of indicators to maintain logical consistency in the evaluation framework. Among our indicators, the old-age dependency ratio represents a reverse indicator where higher values indicate greater societal burden rather than positive outcomes. We applied reverse transformation using Equation 2:


$$X_{\mathrm{ij}}^{’}=\max(X_{j})-X_{\mathrm{ij}}+\min(X_{j})$$ (2)

where *X_ij_* represents the value of indicator *j* for region *i*, and *X’_ij_* is the standardized value.

This transformation preserves the data’s relative structure while reversing its direction, so that the indicators contribute positively to the comprehensive evaluation score. For forward indicators (positive direction), standard min-max normalization was applied, as given in Equation 3:


$$X_{\mathrm{ij}}^{’}=\frac{X_{\mathrm{ij}}-\min(X_{j})}{\max(X_{j})-\min(X_{j})}$$ (3)

The entropy weight calculation then proceeds through systematic steps. We calculate the proportion of each region’s indicator value is calculated using Equation 4:


$$P_{\mathrm{ij}}=\frac{X_{\mathrm{ij}}^{'}}{\sum_{i=1}^{n}X_{\mathrm{ij}}^{,}}$$ (4)

The entropy value for each indicator j is then computed using Equation 5:


$$E_{j}=-k\times \sum_{i=1}^{n}\;P_{\mathrm{ij}}\times \ln P_{\mathrm{ij}}$$ (5)

where $k=\frac{1}{\ln(n)}$ serves as a normalizing constant ensuring $E_{j}\in [0,1]$ .

The information utility value, representing the indicator’s discriminatory power, is obtained from Equation 6:


$$D_{j}=1-E_{j}$$ (6)

The weight for each indicator is determined by Equation 7:


$$W_{j}=\frac{D_{j}}{\sum_{j=1}^{m}D_{j}}$$ (7)

The comprehensive evaluation scores are calculated as weighted sums, following Equation 8:


$${\text{Score}}_{i}=\sum_{j=1}^{m}W_{j}\times X_{\mathrm{ij}}^{’}$$ (8)

This method yields objective, data-driven weights that reflect each indicator’s actual contribution to distinguishing between regions, providing robust composite measures for both population aging characteristics and healthcare resource availability.

The comprehensive evaluation scores calculated using the entropy weight method range from [0, 1]. This index is derived from the weighted sum of standardized original data, where 0 indicates that the province ranks at the lowest level across all evaluation indicators in the sample, and 1 indicates the highest level. Specifically, the theoretical interpretations of the aging comprehensive index and healthcare resource comprehensive index in this study are as follows: scores <0.2 represent low level, 0.2–0.4 represent medium-low level, 0.4–0.6 represent medium level, 0.6–0.8 represent medium-high level, and >0.8 represent high level. This classification standard follows commonly used categorization methods for comprehensive evaluation indices in existing research.

#### 3.3.3 Principal component analysis (PCA)

For provincial-level multidimensional evaluation, we employed principal component analysis to reduce data dimensionality while preserving maximum variance. Principal component analysis implementation followed standardized procedures with rigorous validation. Prior to analysis, all variables underwent z-score standardization (mean = 0, SD = 1) to allow equal contribution potential. We verified sampling adequacy through Kaiser-Meyer-Olkin (KMO) tests, achieving values of 0.827 for the population aging indicators and 0.891 for healthcare resources, both exceeding the 0.8 threshold for “meritorious” factorability. Bartlett’s test of sphericity yielded *p* < 0.001 for both variable sets, confirming sufficient inter-variable correlations.

Component extraction employed the correlation matrix method with Varimax orthogonal rotation to maximize interpretability while maintaining orthogonality. Retention criteria followed dual standards: eigenvalues >1.0 (Kaiser criterion) and cumulative variance explained >80%, with scree plot examination for confirmation. For the population aging indicators, two components met retention criteria (eigenvalues: 2.313, 1.815; cumulative variance: 82.57%). Healthcare resource indicators similarly yielded two components (eigenvalues: 3.123, 2.417; cumulative variance: 92.33%). Rotation converged in 3 iterations for both analyses. Component stability was validated through split-half reliability testing (Cronbach’s α > 0.85) and temporal consistency checks across 5-year subperiods. Component scores were calculated using the regression method given in Equation 9:


$$F_{\mathrm{ik}}=\sum_{j=1}^{p}\alpha_{\mathrm{kj}}\times Z_{\mathrm{ij}}$$ (9)

where *F_ik_* represents the score of province i on component k, *α_kj_* is the component loading of variable *j* on component *k*, and *Z_ij_* is the standardized value of variable *j* for province *i*. Final comprehensive scores were computed as variance-weighted combinations, as in Equation 10:


$${\text{Comprehensive Score}}_{i}=\frac{\sum_{K=1}^{r}[G_{k}\times F_{\mathrm{ik}}]}{\sum_{k=1}^{r}G_{k}}$$ (10)

where G_*k*_ represents the eigenvalue of component k. These scores were subsequently normalized to a 0–1 scale using min-max transformation for integration into coupling coordination degree calculations.

Our analytical framework employs a hierarchical approach where the Entropy Weight Method and PCA serve complementary but distinct purposes. The Entropy Weight Method is applied at the national level to objectively determine indicator weights based on information content across all provinces and years (n = 713 observations), generating weights that reflect each indicator’s discriminatory power in the national context. These national weights support temporal consistency in evaluation criteria.

In contrast, PCA is employed at the provincial level for each province-year combination to reduce dimensionality while preserving maximum variance in the multidimensional data. This dual-method approach leverages the strengths of both techniques: entropy weighting provides objective, data-driven weights for national comparison, while PCA captures province-specific patterns and relationships among variables.

The Pearson correlation coefficient between PCA and entropy method scores was 0.94 (*p* < 0.001), indicating high consistency between the two methods.

#### 3.3.4 Coupling coordination degree model

The quantification of spatiotemporal coupling coordination between population aging and healthcare resources required development of a sophisticated coordination model capable of capturing both individual system development and inter-system balance. We adapted the coupling coordination degree model, originally developed for analyzing economy-environment interactions, to assess the complex interdependencies between the needs of the older population and healthcare resource supply.

The coupling coordination degree model integrates three hierarchical components. First, the coordination index *C_i_* measures the relative balance between the two systems, as defined in Equation 11:


$$C_{i}={\left\{\frac{f(x_{i})\times g(y_{i})}{{\left[\frac{f(x_{i})+g(y_{i})}{2}\right]}^{2}}\right\}}^{k}i=1,2,\ldots ,31$$ (11)

where *f(x_i_)* represents the comprehensive evaluation score for population aging in region i, *g(y_i_)* represents the healthcare resource score, and k = 2 serves as an adjustment coefficient that amplifies coordination differences. This formulation captures the geometric–arithmetic mean ratio, with values approaching 1 indicating balanced development and values near 0 suggesting severe imbalance.

Second, the comprehensive development index T_*i*_ represents the overall development level of both systems, as defined in Equation 12:


$$T_{i}=\mathrm{\alpha f}(x_{i})+\mathrm{\beta g}(y_{i})$$ (12)

where α and β are weight coefficients. We set *α* = *β* = 0.5, reflecting equal importance of population aging characteristics and healthcare resources in determining overall system development.

The Comprehensive Development Index employs equal weights (*α* = *β* = 0.5) to combine the degree of aging and healthcare resource supply level, with the following theoretical justifications:

The coupling coordination degree model adopted in this study originates from coupling theory in physics, emphasizing the coordinated interactive relationship between two subsystems. Under the coupling coordination analysis framework, equal weighting is standard practice, indicating that the research focuses on the coupling coordination degree between two subsystems rather than the dominance of either subsystem.

From a health policy perspective, the degree of aging represents the demand side of healthcare services, while healthcare resource supply represents the supply side. Both are equally important in health system planning. Equal weighting reflects the policy goal of supply–demand balance and aligns with the core requirement in the “Healthy China 2030” strategy for healthcare resource allocation to match population structure.

Equal weighting is widely adopted in similar studies: coupling coordination analyses of healthcare resource supply, demand and change in the older population in China, and of medical resources for older adults in the Yellow River Basin, both use equal weights (16, 17), which supports the applicability and comparability of this setting.

To verify the robustness of equal weighting, we conducted sensitivity analyses under different weight combinations. When weight combinations were set as α:β = 0.4:0.6 (resource-weighted), 0.5:0.5 (equal weights), and 0.6:0.4 (aging-weighted), the national mean coupling coordination degrees were 0.502, 0.487, and 0.471, respectively. The Spearman correlation coefficients of provincial rankings under the three weight settings were all greater than 0.95, indicating highly stable inter-provincial relative rankings. Furthermore, regardless of the weight combination adopted, the basic pattern of eastern regions having the highest coupling coordination degrees and western regions having the lowest remained unchanged, and main research conclusions were not affected by weight settings. This indicates that equal weighting is robust in this application and the reliability of research conclusions.

The coupling coordination degree *D_i_* synthesizes coordination and development, as given in Equation 13:


$$D_{i}=\sqrt{C_{i}\times T_{i}}$$ (13)

This geometric mean formulation ensures that high coupling coordination degrees require both good coordination (balanced development) and high overall development levels. The resulting D_i_ values range from 0 to 1, with higher values indicating better spatiotemporal coupling coordination.

The five-level classification of coupling coordination degrees was established through a combination of statistical analysis and theoretical considerations. We employed Jenks natural breaks optimization, an established clustering algorithm that minimizes within-class variance while maximizing between-class variance, to identify statistically meaningful thresholds in the empirical distribution of coupling coordination degrees across all province-year observations (n = 713). The algorithm identified natural breakpoints at 0.196, 0.298, 0.401, and 0.598, which we rounded to 0.20, 0.30, 0.40, and 0.60 for interpretability. These statistically-derived thresholds align with theoretical frameworks from coordination theory and WHO’s health system performance categories, resulting in five categories: insufficient coordination (D < 0.20), coordination to be improved (0.20 ≤ D < 0.30), optimization zone (0.30 ≤ D < 0.40), good coordination (0.40 ≤ D < 0.60), and high coordination (D ≥ 0.60).

Four boundaries on the interpretation of the coupling coordination degree should be stated explicitly. First, the model is not a causal model. Equations 11–13 are deterministic transformations of two composite indices; no parameter is estimated, no counterfactual is defined, and no claim is made that a change in one subsystem produces a change in the other. Second, the model does not measure the matching of healthcare demand to healthcare supply. Both subsystems are composite indices built from provincial aggregates, and the aging index summarizes demographic characteristics, not measured healthcare need; the coordination degree therefore describes the correspondence between two statistical constructs, not the extent to which the healthcare requirements of older adults are met. Third, the index is not specific to older adults on the supply side. The six capacity indicators refer to the whole healthcare system, so a high coordination degree is consistent with an absence of geriatric beds, long-term care facilities, and geriatricians, none of which is observable in provincially consistent form for 2000–2023. Fourth, the classification thresholds are internal to the sample. They were obtained by natural breaks optimization on the pooled distribution of D across 713 province-year observations and rounded for legibility; they describe relative position within the observed distribution and carry no external standard of adequacy. Taken together, a high coupling coordination degree is a necessary but not a sufficient condition for adequate healthcare provision for older adults, and the analysis that follows should be read as descriptive and diagnostic rather than evaluative.

### 3.4 Statistical analysis

The analysis is descriptive and index-based, and is applied to a panel of 31 provinces observed over 24 years. Data quality assurance involved systematic screening for missing values, outliers and implausible entries. Missing values (31 of 744 province-year observations, 4.2%, confined to Tibet and Xinjiang in 2000–2003) were imputed by linear interpolation between the nearest available years, as set out in Section 3.1; this is the only imputation procedure applied in the study. No test of distributional form was carried out on the indicator series, and descriptive results are therefore reported without a parametric versus non-parametric split and without group comparison tests: the distribution of every indicator across the pooled panel is summarized in Table 2, national values by period in Table 3, cluster-wise ranges across all province-years in Table 4, and inter-provincial dispersion is reported as the coefficient of variation. Principal component analysis was implemented in SPSS 21.0 and assessed through Kaiser-Meyer-Olkin measures (>0.8) and Bartlett’s test of sphericity (*p* < 0.001), with split-sample checks between eastern and western provinces. Component scores underwent min-max normalization to generate 0–1 scales for the coupling coordination degree calculations. Agreement between the entropy and PCA composites was summarized by Pearson correlation (*r* = 0.94), the association between the aging and healthcare composites by Pearson correlation, and the stability of provincial rankings under alternative weights by Spearman correlation (Section 3.3.4). These correlations are reported as descriptive summaries of association. No regression model, no fixed- or random-effects panel estimator and no spatial econometric model was fitted. The coupling coordination degree is a deterministic transformation of the composite indices (Equations 11–13) rather than an estimated parameter, so the panel structure is used to describe evolution over time rather than to identify effects. Global and local statistics of spatial autocorrelation, such as Moran’s I and local indicators of spatial association (21), were likewise not estimated: the spatial results reported below rest on mapping and Jenks natural-breaks classification in ArcGIS 10.8, using colourblind-friendly palettes. Consequently, the differences described in the Results are observed differences within the study sample and are not tested against a null hypothesis, and the absence of panel estimation and of spatial statistical testing is stated as a limitation in Section 5.4 and as a priority for future work.

**Table 2:** Descriptive statistics of the population aging and healthcare resource indicators, Chinese provinces, 2000–2023 (*n* = 713 province-years).

Subsystem	Indicator	Mean	SD	Median	IQR (P25–P75)	Min	Max
Population Aging Subsystem	Population aged ≥65 years (10,000 persons)	468.72	321.45	382.16	201.33–647.91	12.68	1624.37
	Density of population aged ≥65 (persons/km^2^)	62.37	91.52	26.84	8.19–73.52	0.41	426.83
	Aging rate (%)	10.14	3.16	9.70	7.60–12.50	4.20	20.00
	Old-age dependency ratio (%)	16.83	5.79	15.92	12.31–20.77	6.45	34.12
	Life expectancy at birth (years)	73.56	4.12	73.81	70.15–77.63	64.37	84.11
Healthcare Resource Subsystem	Number of hospitals	316.22	204.58	273.00	146.00–421.00	12.00	987.00
	Hospital beds (10,000)	16.43	10.25	14.18	8.36–22.74	0.43	68.59
	Hospital health technicians (10,000 persons)	10.72	6.81	9.46	5.27–14.83	0.29	41.26
	Number of primary healthcare institutions	21658.34	14962.75	18241.00	8,956–30,174	864.00	86742.00
	Primary healthcare beds (10,000)	4.16	2.73	3.52	1.86–5.64	0.06	16.31
	Primary healthcare technicians (10,000 persons)	3.95	2.57	3.41	1.69–5.40	0.05	14.27
Composite Index	Aging comprehensive score (0–1 normalized)	0.472	0.215	0.451	0.306–0.637	0.084	0.926
	Healthcare resource comprehensive score (0–1 normalized)	0.369	0.247	0.312	0.153–0.548	0.006	1.000
Coupling Coordination	Coupling coordination degree D (0–1 normalized)	0.487	0.203	0.465	0.331–0.642	0.000	1.000

*n* = 713 province-year observations from 31 provincial-level administrative regions over 2000–2023 (theoretical n = 744; 31 observations imputed by linear interpolation, Section 3.1). SD, standard deviation; IQR, interquartile range (25th–75th percentile). The comprehensive scores and the coupling coordination degree D are dimensionless and min–max normalized over the pooled sample, so that values of 0 and 1 denote the sample minimum and maximum rather than an absence of coordination or perfect coordination.

**Table 3:** Summary of temporal evolution in China.

Period	Aging rate change	Healthcare score change	Annual growth rate	Key characteristics
2000–2010	7.0% → 8.9%	0.02 → 0.109	Aging: 0.19%/year; Healthcare: 0.009/year	Slow growth phase; initial healthcare reforms
2010–2023	8.9% → 14.9%	0.109 → 0.91	Aging: 0.46%/year; Healthcare: 0.062/year	Rapid acceleration; comprehensive reform implementation

**Table 4:** Provincial aging and healthcare resource distribution.

Region	Representative provinces	Aging coefficient (%)	Healthcare score	Spatial characteristics
Northeast Cluster	Liaoning, Jilin, Heilongjiang	8.7–20.0	0.25–0.58	From moderate to super-aged; limited resource growth
Eastern Coast	Shanghai, Jiangsu, Zhejiang	8.4–18.7	0.20–0.75	Rapid aging; expanding resources
Central Belt	Henan, Hubei, Hunan	7.8–15.8	0.42–1.00	Accelerating aging; substantial resource improvement
Western Periphery	Tibet, Qinghai, Xinjiang	4.2–10.0	0.006–0.29	Slowest aging; persistently scarce resources

Values are the cluster-wise minimum and maximum across all province-years from 2000 to 2023, and not single-year values. For the Western Periphery cluster, the lower bound of the Healthcare Score, 0.006, corresponds to Tibet in 2000; Tibet’s 2023 score of 0.021 therefore lies within this range, close to its floor.

The distribution of each individual indicator across the pooled panel is reported before any aggregation is undertaken, so that the composite indices and the coupling coordination degree can be read against the raw scales from which they are derived. Table 2 summarizes the mean, standard deviation, median, interquartile range and observed range of the five population aging indicators, the six healthcare resource indicators, the two normalized composite scores and the coupling coordination degree across the 713 valid province-year observations (31 provinces, 2000–2023). The dispersion statistics quantify the heterogeneity that motivates the analysis: for several indicators the standard deviation approaches or exceeds the mean and the maximum exceeds the minimum by two orders of magnitude, and for every indicator the mean lies above the median, indicating right-skewed distributions in which a small number of populous eastern province-years sit far above the bulk of the sample. The medians and interquartile ranges are therefore reported alongside the means and standard deviations throughout, and the descriptive, non-parametric character of the analysis set out above follows from these distributional features.

Because 31 of the 744 province-year cells (4.2%) were completed by linear interpolation, and these cells are confined to Tibet and Xinjiang for the years 2000 to 2003, we examined whether the reported patterns depend on the imputed values. Four checks were carried out. We re-derived the national temporal series and the spatial distribution with Tibet and Xinjiang removed from the panel, which excludes every imputed value. The trajectory of the national aging coefficient and of the composite indices, together with the east–west gradient across the remaining 29 provinces, was compared with the full-sample result. We re-imputed the 31 cells by three alternative schemes, namely carrying the nearest observed value, cubic spline interpolation, and province-specific linear extrapolation, and recomputed the composite indices and the coupling coordination degree under each. The province-year coordination values and rankings were then compared with the linear-interpolation baseline using the Pearson and Spearman coefficients and the maximum absolute deviation in D. We replaced each imputed value with the lower and the upper value of its two nearest observed years and checked whether the aging-stage classification and the coordination-level classification of the two provinces remained unchanged across these bounds. Because the entropy weights are derived at the national level and the two affected provinces carry a small share of the national population, we quantified the change in the national normalized composite scores for 2000 to 2003 when the imputed cells were moved to their bounds.

Across all four checks the reported patterns are preserved: the national trajectories and the east–west gradient are unchanged after excluding Tibet and Xinjiang (Supplementary Table S1); the coordination degree is almost identical under three alternative imputations (Pearson *r* = 0.972–0.981, Spearman *ρ* = 0.965–0.974, maximum |Δ*D*| ≤ 0.016; Supplementary Table S2); the aging-stage and coordination-level classifications of both provinces do not change under the lower/upper bounds (Supplementary Table S3); and the national normalized composite scores for 2000–2003 shift by at most ±0.006 (Supplementary Table S4).

## 4 Results

### 4.1 Temporal evolution of population aging

The longitudinal analysis of China’s population aging from 2000 to 2023 indicates a substantial demographic transformation characterized by accelerating aging rates and distinct temporal phases. China’s national aging coefficient increased from 7.0% in 2000 to 14.9% in 2023, doubling over the 24-year study period. This trajectory exhibits a two-stage pattern of “acceleration-stabilization” that reflects the complex interplay of demographic policies, socioeconomic development, and epidemiological transitions.

During the low-speed growth period spanning 2000 to 2010, the aging coefficient exhibited a relatively modest increase from 7.0 to 8.9%, translating to an annual average growth of 0.19 percentage points. The year 2000 marked a pivotal demographic milestone as China officially crossed the internationally recognized threshold of a 7% share of the population aged 65 and above, formally entering the ranks of aging societies. This initial phase of population aging was predominantly driven by sustained fertility decline, with the total fertility rate further decreasing from an already low 1.22 in 2000 to 1.18 by 2010. However, the aging rate during this period remained relatively constrained due to the substantial buffering effect provided by the large cohorts born during the 1960s-1980s who were still in their prime working ages. This demographic dividend period, characterized by a favorable ratio of working-age to dependent populations, effectively moderated the pace of population aging despite ongoing fertility decline. Additionally, the gradual nature of aging during this phase allowed for some degree of institutional adaptation, though as subsequent analysis indicates, healthcare resource adjustments lagged behind demographic changes.

China’s demographic and healthcare landscape underwent substantial transformation from 2000 to 2023. The national aging coefficient doubled from 7.0 to 14.9%. Over the same period the normalized composite healthcare score rose from 0.02 to 0.91, a relative movement within a bounded index rather than a proportional increase in the physical quantity of healthcare resources. In absolute terms, hospital beds reached 9.10 million by 2023, approximately 6.4 beds per 1,000 population, and licensed physicians increased from 2.41 million in 2010 to 4.28 million in 2023, an increase of 77.6% and approximately 3.0 physicians per 1,000 population. Table 3 summarizes these key transitions.

The rapid acceleration period from 2010 to 2023 saw a marked intensification of the aging process, with the national aging coefficient rising to 13.5% by 2020 and further climbing to 14.9% by 2023. This represents an annual average increase of 0.46 percentage points, marking a 2.4-fold acceleration compared to the previous decade. The year 2010 emerges as a critical inflection point in China’s demographic trajectory, after which the aging process entered a more rapid phase. This acceleration resulted from the convergence of multiple demographic forces operating simultaneously. Population structure inertia accumulated over previous decades began to manifest more prominently as the large birth cohorts from the 1950s and 1960s progressively entered the older age categories. The lagged effects of stringent birth control policies also implemented since the late 1970s became increasingly apparent as smaller younger cohorts failed to numerically balance the expanding older population. Continuous improvements in life expectancy, in addition, rising from 71.4 years in 2000 to 78.2 years in 2023, not only increased the absolute number of older adults but also extended the duration of old age, amplifying the aging coefficient from both directions (Figure 2).

**Figure 2:**
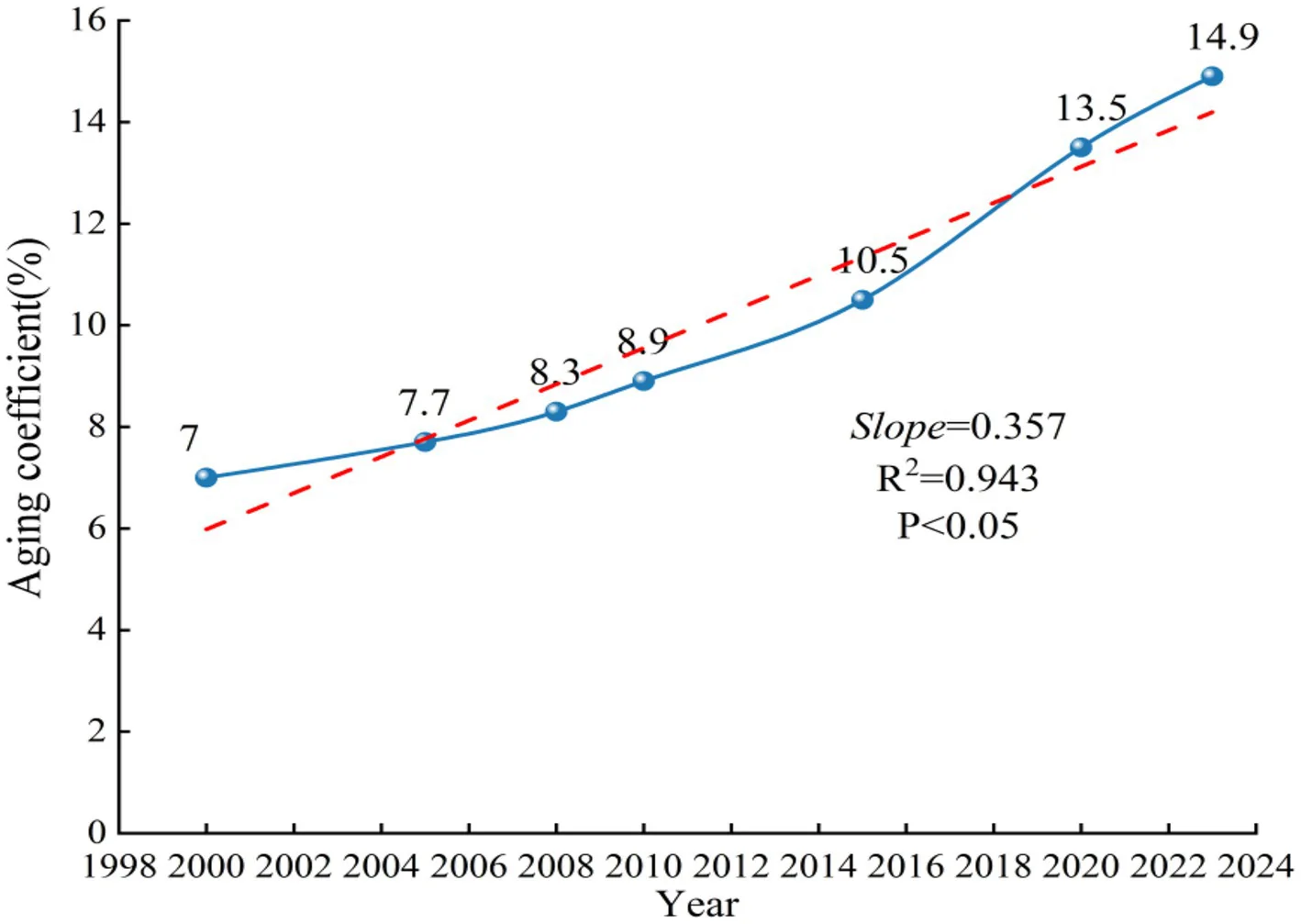
Population aging trends of China from 2000 to 2023.

The temporal evolution also reveals important sub-period variations within these broader phases. Between 2010 and 2015, the aging coefficient increased by 1.6 percentage points, while the subsequent period from 2015 to 2020 witnessed an even steeper increase of 3 percentage points. This acceleration within the acceleration phase suggests that China’s population aging is following a non-linear trajectory with accelerating growth in some periods. The most recent data from 2020 to 2023 shows a slight moderation in the growth rate, potentially indicating the beginning of a new phase or temporary stabilization. However, demographic projections based on current age structures suggest this moderation is likely temporary, with aging expected to resume rapid growth as even larger cohorts approach the older age thresholds in the coming decade.

### 4.2 Spatial evolution of population aging

The spatial distribution of population aging across China’s 31 provincial-level administrative units reveals pronounced geographic heterogeneity and evolving spatial patterns that reflect the complex interplay of regional development disparities, migration dynamics, and policy variations. Throughout the 2000–2023 study period, aging exhibited a persistent east–west gradient overlaid with multi-center clustering patterns, creating a spatially complex mosaic of demographic transitions occurring at different paces and intensities across the national territory (Figure 3).

**Figure 3:**
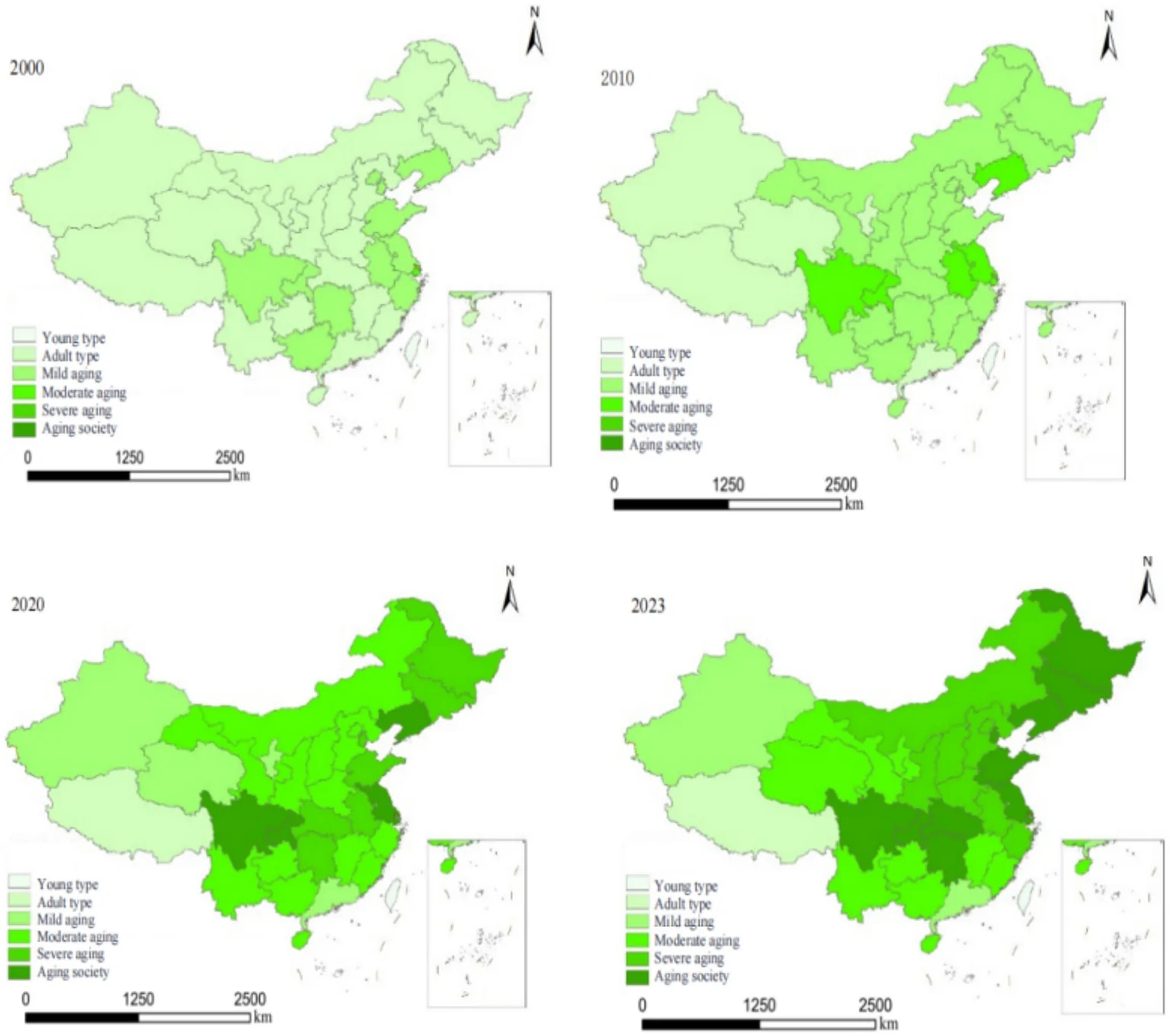
Spatial distribution of the aging population coefficient in Chinese provinces from 2000 to 2023.

In 2000, when China’s national aging coefficient reached 7.0%, the spatial distribution already displayed clear regional differentiation. The eastern and northeastern provinces demonstrated advanced demographic transition, with Shandong achieving 8.8%, Shanghai reaching 11.5%, Jiangsu attaining 9.5%, Zhejiang recording 9.2%, Liaoning showing 8.7%, Beijing registering 8.4%, and Tianjin displaying 8.6%, all having progressed beyond the 7% threshold into mild aging societies. Shanghai stood out as the one province in the sample that had already entered moderate aging above 10%, presaging the rapid aging that would later characterize the entire eastern seaboard. Central provinces showed mixed patterns, with Anhui at 8.1%, Sichuan at 8.9%, Chongqing at 8.5%, Hunan at 8.2%, and Guangxi at 7.8% joining the aging society ranks, while others remained below the threshold. The western regions presented a starkly different demographic landscape, with provinces like Tibet maintaining 4.2%, Xinjiang sustaining 5.8%, and Qinghai preserving 5.5%, creating an aging coefficient range of 7.3 percentage points between the highest and lowest provinces (Table 4).

Having established the temporal dynamics at the national level, we now examine how these patterns manifest spatially across provinces. The spatial evolution reveals persistent heterogeneity with emerging clustering patterns.

By 2010, the spatial pattern had evolved to show more pronounced aging centers while maintaining the fundamental east–west gradient. The Yangtze River Delta-Chengdu-Chongqing axis emerged as a continuous moderate aging belt, with Shanghai reaching 13.3%, Jiangsu achieving 12.0%, Anhui attaining 10.8%, Sichuan recording 11.0%, and Chongqing displaying 11.5%, all exceeding 10% aging coefficients. This trans-regional aging corridor reflected both the natural progression of demographic transition in economically developed areas and the specific impacts of stringent family planning policy implementation in these regions. Northeast China began showing accelerated aging, with Liaoning advancing to 10.8%, foreshadowing the severe aging that would later characterize the entire northeastern region. The persistence of young age structures in western provinces like Tibet at 5.0%, Xinjiang at 6.8%, and Qinghai at 6.5% during this period reflected not only different demographic starting points but also variations in policy implementation and socioeconomic development levels.

The 2023 spatial distribution represents a mature phase of differential aging across China, with the national aging coefficient reaching 14.9% but displaying pronounced provincial variations. The northeastern provinces emerged as the most aged region in the sample, with all three provinces, Liaoning at 20.0%, Jilin at 18.5%, and Heilongjiang at 17.8%, exceeding 17.5%, effectively forming a super-aged northeast cluster. This severe aging in the northeast reflects the compound effects of early industrialization leading to early demographic transition, massive out-migration of working-age populations seeking opportunities elsewhere, and harsh climatic conditions potentially affecting fertility and mortality patterns. The Yangtze River Delta maintained its position as a major aging center, though with interesting internal variations: while Shanghai at 18.7% and Jiangsu at 17.3% showed severe aging, Zhejiang displayed relatively moderate levels at 15.2%, possibly due to its more balanced urban–rural structure and continued economic dynamism attracting younger migrants.

The Chengdu-Chongqing region in western China emerged as an unexpected aging hotspot, with Chongqing at 17.8% and Sichuan at 17.1% surpassing many eastern provinces in aging intensity. This phenomenon challenges simplistic east–west aging narratives and highlights the role of specific regional factors, including historically high urbanization rates in these municipalities, early adoption of family planning policies, and significant out-migration from surrounding rural areas. Central provinces displayed heterogeneous patterns, with some like Anhui at 15.8% and Hubei at 15.3% showing substantial aging, while others like Henan at 13.9% remained below the national average despite large older populations in absolute terms.

Notably, several provinces exhibited aging patterns that deviated from regional norms, providing insights into the complex determinants of spatial aging patterns. Guangdong at 10.2% and Fujian at 11.8%, despite their eastern coastal locations, maintained relatively low aging coefficients due to sustained in-migration of younger workers drawn by manufacturing and service sector opportunities. Conversely, Gansu in the northwest showed higher-than-expected aging at 12.5%, reflecting economic stagnation and youth out-migration. The persistence of young age structures in Tibet at 6.8%, Xinjiang at 9.2%, and Qinghai at 10.0% through 2023, while narrowing the gap with national averages, continues to reflect these regions’ distinctive demographic, cultural, and policy contexts. The overall spatial pattern by 2023 thus presents a complex mosaic where traditional east–west gradients intersect with urban–rural differentials, migration effects, and local policy variations to create a highly differentiated aging landscape requiring equally differentiated policy responses.

### 4.3 Temporal evolution of healthcare resources

The temporal trajectory of China’s healthcare resource development from 2000 to 2023 shows a substantial expansion, with the national comprehensive healthcare resource score rising from 0.02 to 0.91 on the min–max normalized scale. Because this index is bounded, the change is a relative movement within the index rather than a proportional increase in physical resources; in absolute terms, hospital beds reached 9.10 million by 2023, about 6.4 per 1,000 population, and licensed physicians reached 4.28 million. The series is reported here without attribution to particular causes. Policy priorities, fiscal commitments and demographic pressure all changed over the same period, and they are discussed as context in Section 5.2 rather than as tested explanations. What the series does show is a set of distinct phases, described below (Figure 4).

**Figure 4:**
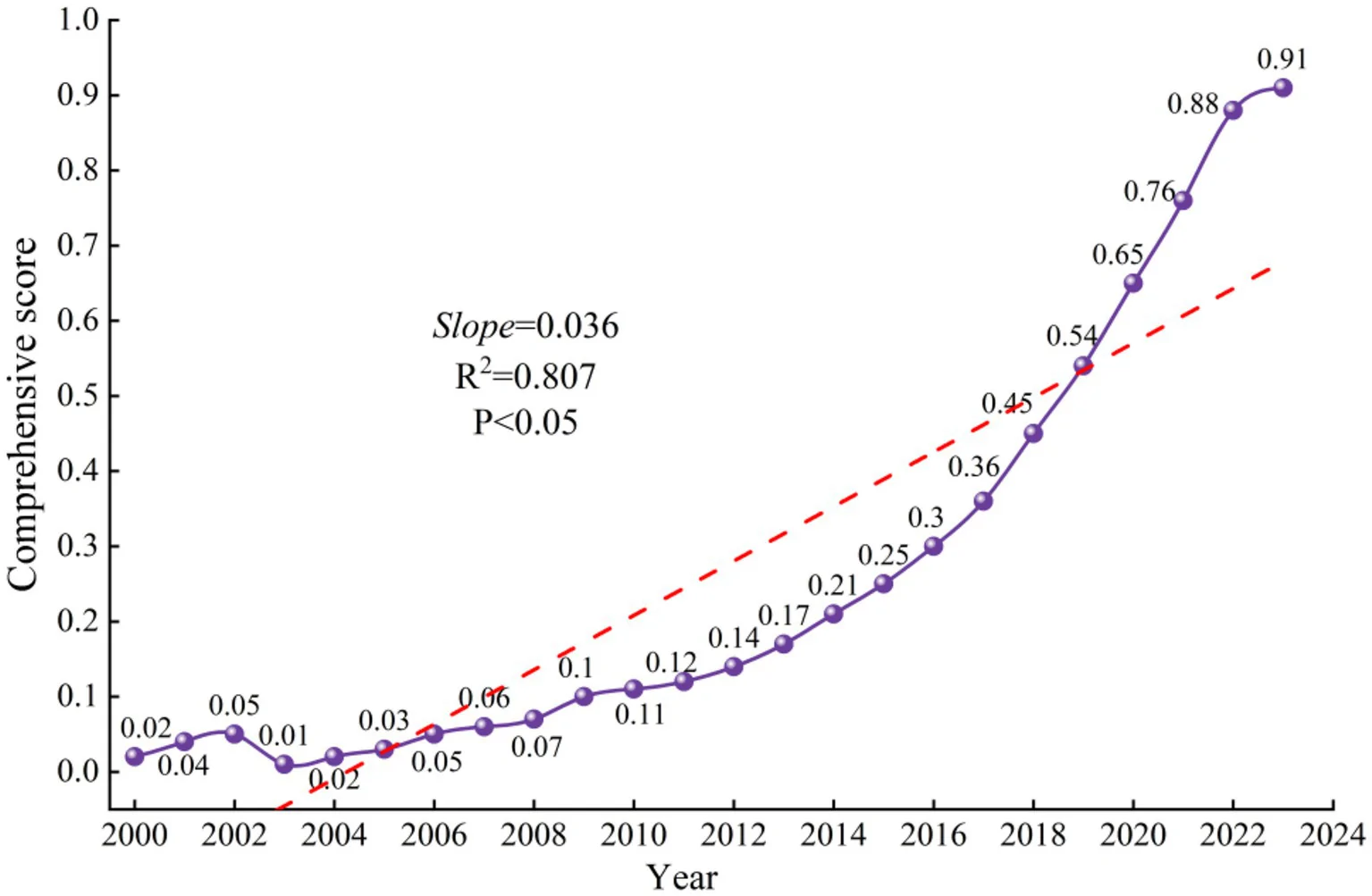
Trends in the development level of medical and health resources in China from 2000 to 2023.

The low-speed accumulation period from 2000 to 2010 was characterized by modest but foundational healthcare resource development. Starting from a notably low base of 0.02 in 2000, the comprehensive score reached 0.109 by 2010, representing an annual average increment of 0.009 points. Over this decade the government share of total health expenditure fell from 38.3% in 2000 to 28.7% in 2005 before recovering (China Health Statistical Yearbook). The coincidence with the early stage of market-oriented reform is noted, and no effect of those reforms on the series was estimated. The period witnessed the initial establishment of the New Cooperative Medical Scheme (NCMS) in rural areas starting in 2003, though its resource implications remained limited during this phase due to low reimbursement rates and narrow coverage scope. Hospital resources during this period grew at a moderate pace, with the number of hospital beds increasing from 3.18 million to 4.79 million nationally, representing a 50.6% growth that barely kept pace with population growth and urbanization. Primary healthcare institutions faced particular challenges during this phase, with many township health centers and village clinics experiencing resource constraints due to fiscal decentralization policies that shifted funding responsibilities to local governments with limited revenue bases.

The rapid expansion period from 2010 to 2023 marked a shift in healthcare resource development, characterized by higher growth rates and structural improvements. The comprehensive score accelerated from 0.109 in 2010 to 0.757 in 2020, before reaching the normalized maximum of 0.91 in 2023. The annual average increase of 0.062 points during this period represents a seven-fold acceleration compared to the previous decade, reflecting fundamental policy reorientations and increased resource commitments. The year 2009 emerges as a critical juncture with the launch of comprehensive healthcare reforms aimed at achieving universal health coverage, though the resource implications became most apparent from 2010 onward. Government health expenditure surged from 498.3 billion RMB in 2010 to 2,089.1 billion RMB in 2020, a 4.2-fold increase that far exceeded GDP growth rates. This period witnessed massive infrastructure development, with hospital beds reaching 9.1 million by 2023, while the number of licensed physicians grew from 2.41 million to 4.28 million, representing a 77.6% increase that finally began addressing long-standing physician shortage issues.

The 2010–2023 expansion phase itself exhibited important sub-period variations that illuminate the evolving priorities and challenges in healthcare resource allocation. The 2010–2015 sub-period focused primarily on coverage expansion and basic infrastructure development, with particular emphasis on strengthening primary healthcare networks. The number of community health service centers in urban areas increased by 68%, while township health centers received substantial upgrades in equipment and staffing. The 2015–2020 sub-period shifted focus toward quality improvement and service integration, marked by the initiation of hierarchical diagnosis and treatment systems and the promotion of medical alliances. This period also witnessed accelerated development of specialized care resources for older adults, including the establishment of geriatric departments in secondary and tertiary hospitals and the pilot implementation of integrated medical and long-term care facilities for older adults.

The COVID-19 pandemic period (2020–2023) induced another transformation in healthcare resource development patterns. The pandemic exposed critical weaknesses in public health infrastructure and emergency response capabilities, leading to extraordinary resource mobilization and system strengthening efforts. Public health expenditure increased by 43% between 2019 and 2021, while investments in infectious disease hospitals, intensive care units, and emergency response systems increased markedly. The rapid development of internet hospitals and telemedicine platforms during this period, growing from 119 in 2018 to over 1,700 by 2023, represents a technological advance that expanded healthcare resource accessibility. This digital transformation particularly benefited older adults in remote areas, partially compensating for physical resource disparities. The normalized score of 0.91 achieved in 2023 thus represents not only quantitative resource abundance relative to historical levels but also qualitative improvements in resource allocation efficiency, service integration, and technological sophistication that position the healthcare system to better address the challenges of an aging society.

### 4.4 Spatial evolution of healthcare resources

The spatial distribution of healthcare resources across China’s provinces from 2000 to 2023 reveals a complex geographic pattern characterized by persistent disparities, evolving cluster formations, and gradual but incomplete convergence trends. The fundamental “east-dense, west-sparse” configuration has remained a defining feature throughout the study period, though the specific manifestations and intensity of spatial inequalities have evolved considerably. This spatial evolution reflects the intricate interplay of economic development levels, fiscal capacities, policy priorities, and geographical constraints that shape healthcare resource allocation across China’s vast and diverse territory (Figure 5).

**Figure 5:**
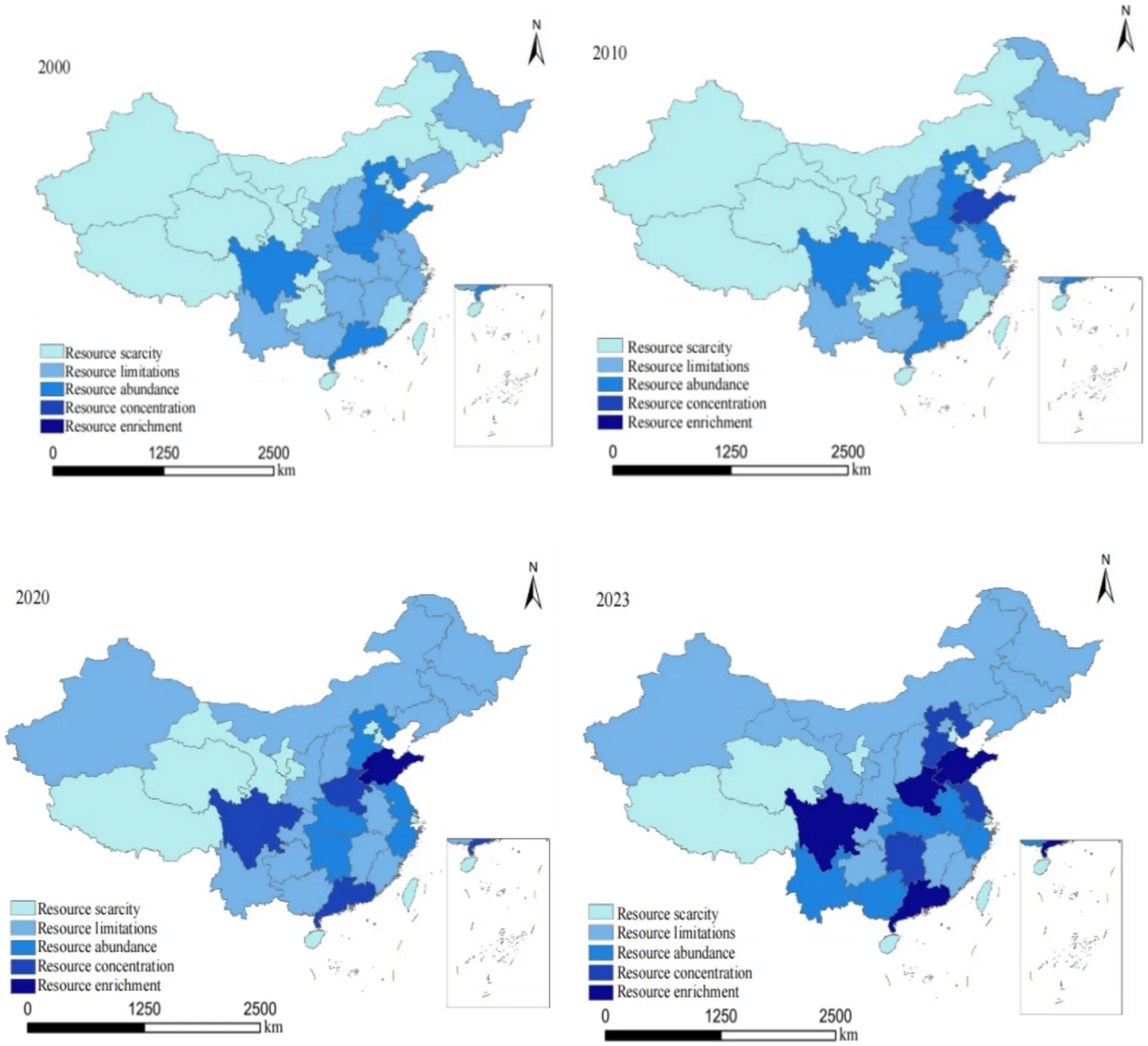
Spatiotemporal evolution pattern of comprehensive scores of provincial medical and health resources in China from 2000 to 2023.

In 2000, the spatial distribution of healthcare resources displayed pronounced polarization, the cross-provincial mean of the normalized healthcare resource scores averaging merely 0.25 and exhibiting a coefficient of variation of 0.68, indicating severe inter-provincial disparities. Only three provinces, Shandong (0.58), Hebei (0.50), and notably Sichuan (0.64), achieved scores placing them in the balanced resource category. Sichuan’s surprisingly high score reflected its historical role as a medical education center in western China and the legacy of Third Front construction that had established substantial healthcare infrastructure in the province. The majority of eastern coastal provinces, despite their economic advantages, scored in the limited resource range (0.21–0.40), with Beijing (0.36), Shanghai (0.33), and Guangdong (0.38) showing moderate levels that reflected their compact territories and high efficiency rather than absolute resource abundance. A notable feature was the vast resource desert spanning western China, with 14 provinces classified as resource-scarce. Tibet (0.006), Qinghai (0.027), and Ningxia (0.015) formed a resource scarcity triangle, with scores approaching zero on the standardized scale. These minimal scores reflected not only low absolute numbers of healthcare facilities and personnel but also the substantial challenges of providing healthcare across sparsely populated, geographically challenging territories with limited transportation infrastructure.

By 2010, the spatial pattern had evolved to show emerging regional clusters while maintaining fundamental disparities. The cross-provincial mean score increased to 0.28, with the coefficient of variation declining slightly to 0.61, suggesting modest convergence. The Yangtze River Delta began emerging as a healthcare resource cluster, with Jiangsu advancing to 0.39 and Zhejiang to 0.33, joining Shanghai in forming a contiguous zone of relatively abundant resources. Central provinces showed notable improvements, with Henan (0.57) and Hunan (0.42) achieving balanced resource status, reflecting the early effects of policies aimed at strengthening healthcare systems in populous central provinces. The western region continued to lag severely, though some improvements were evident. Xinjiang’s score increased to 0.19, approaching the threshold for limited resources, benefiting from central government investments in border region stability and development. However, the Qinghai-Tibet plateau remained virtually unchanged, with Tibet at 0.009 and Qinghai at 0.033, highlighting the substantial challenges of healthcare delivery in high-altitude, low-density regions. The persistence of a 63-fold difference between the highest (Henan) and lowest (Tibet) scores underscored the magnitude of spatial healthcare inequalities.

The year 2023 represents a matured but still unequal spatial configuration of healthcare resources. The cross-provincial mean score reached 0.41, with the coefficient of variation decreasing to 0.52, indicating continued but slow convergence. Three distinct high-resource clusters had crystallized: the Beijing-Tianjin-Hebei metropolitan region (though paradoxically, Beijing’s score of 0.22 and Tianjin’s 0.10 remained relatively low due to their small territories and the normalization method used); the Yangtze River Delta centered on Jiangsu (0.75) and extending to Zhejiang (0.57) and Shanghai (0.20); and the Pearl River Delta anchored by Guangdong (0.87). These clusters are described from the mapped distribution. Spatial spillover was not estimated, since neither a spatial autocorrelation statistic nor a spatial econometric model was computed (Section 3.4), so movement of patients between provinces is raised as a possible reading only. The emergence of a central China resource belt was particularly notable, with Henan achieving the maximum score of 1.00, followed by Hubei (0.57) and Hunan (0.69). This central-region pattern coincided with policies to develop regional medical centres in populous provinces as alternatives to the crowded coastal facilities. Those policies are noted as context and their effect on the series was not tested.

The 2023 spatial pattern also revealed important nuances in resource distribution. Several western provinces showed substantial improvements, with Yunnan (0.45) and Shaanxi (0.41) entering the balanced resource category, benefiting from targeted poverty alleviation programs that included healthcare infrastructure development. The northwest corridor along the Belt and Road showed enhanced resources, with Gansu (0.31) and Xinjiang (0.29) displaying above-expected scores given their economic development levels. However, persistent resource deserts remained, with Tibet (0.021) and Qinghai (0.038) still in the severe scarcity category despite absolute improvements. The 48-fold difference between the highest and lowest scores in 2023 narrowed from earlier levels while remaining wide. It is a difference in normalized index values, and we compiled no comparably constructed indices for other countries against which it might be placed.

Underlying these provincial scores were important intra-provincial disparities that the aggregate data mask. Provinces with high overall scores often displayed pronounced internal inequalities, with resources concentrated in provincial capitals and major cities while rural and mountainous areas remained underserved. For instance, while Guangdong achieved a high provincial score, the Pearl River Delta region accounted for over 70% of the province’s high-level medical resources despite housing only 45% of its population. Similarly, in western provinces, limited resources were typically concentrated in a single major city, leaving vast hinterlands with minimal healthcare infrastructure. These nested spatial inequalities, which run between regions, between provinces and within them, created a complex geography of healthcare access that simple provincial averages only partially capture. The evolution from 2000 to 2023 thus represents not a simple convergence narrative but rather a complex reconfiguration of spatial inequalities operating at multiple scales, shaped by the competing forces of market concentration, policy intervention, and geographical constraints.

### 4.5 Spatiotemporal coupling coordination between aging and healthcare resources

The analysis of spatiotemporal coupling coordination between population aging and healthcare resources from 2000 to 2023 reveals a complex dynamic characterized by overall improvement trajectory punctuated by persistent regional disparities and emerging misalignment patterns. The national coupling coordination degree index progresses from 0.00 in 2000 to 1.00 in 2023. These endpoints are the lower and upper bounds of the min–max normalization applied to the composite scores that enter the coupling coordination model, in which the pooled sample minimum is rescaled to 0.00 and the pooled sample maximum, namely the 2023 national value, to 1.00. They are sample-anchored relative extrema and denote neither an absence of coordination in 2000 nor empirically perfect coordination in 2023. The coupling coordination degree is obtained from the deterministic closed-form expressions given in Section 3.3.4 rather than from a model with estimated parameters, and it is described throughout as normalized to the sample maximum. The temporal account below is therefore stated in terms of the underlying composite scores: the aging score rises as the aging coefficient doubles from 7.0 to 14.9%, while the healthcare composite score rises from 0.02 to 0.91, so that coordination improves from a very low base largely through resource expansion. However, decomposition of this aggregate trend reveals nuanced patterns of convergence and divergence that carry critical implications for policy formulation and resource allocation strategies in China’s rapidly aging society (Figure 6).

**Figure 6:**
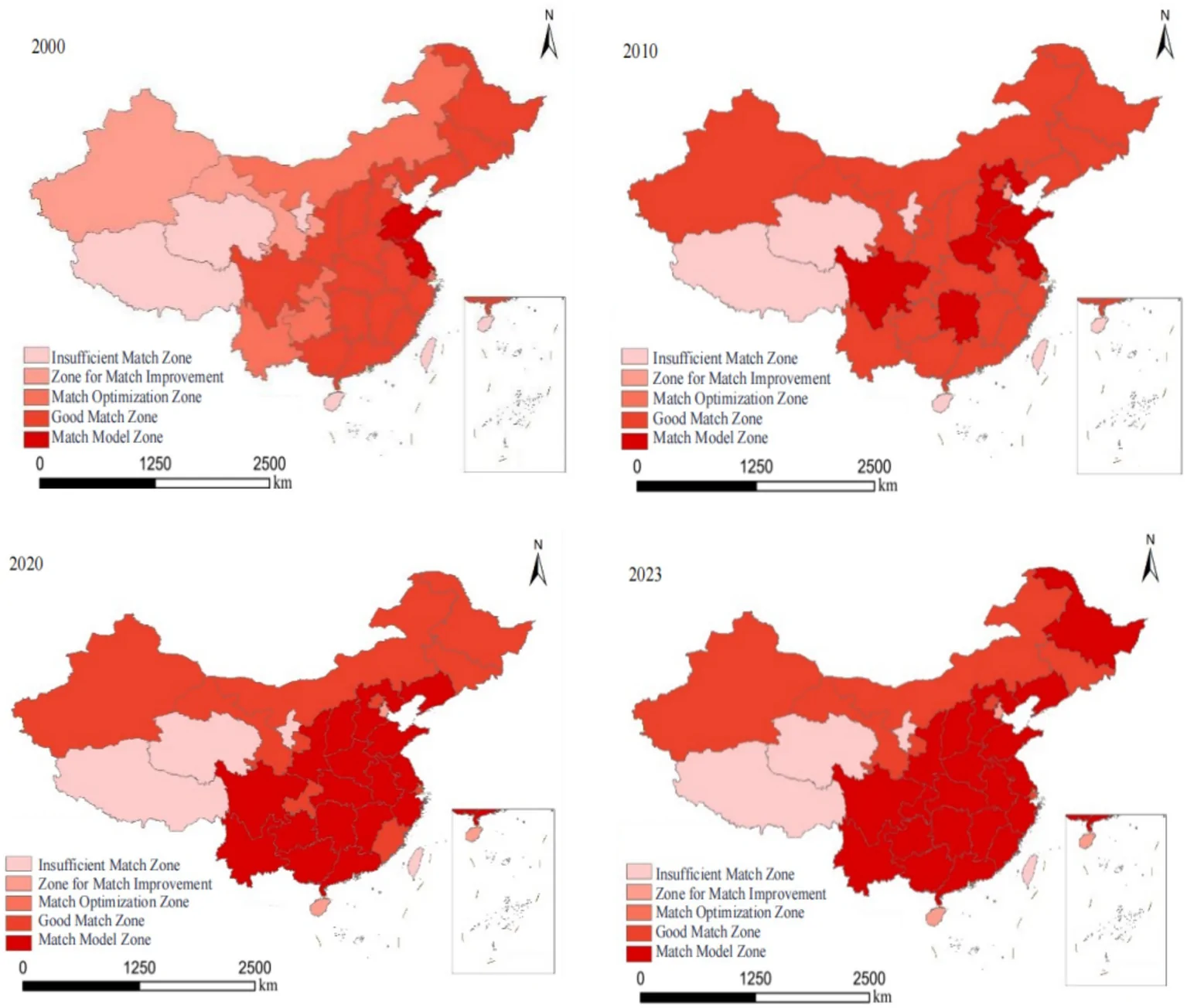
Spatial heterogeneity of the coupling coordination degree between population aging and healthcare resource capacity in China, 2000−2023.

The temporal evolution of coupling coordination degree exhibits two distinct phases with markedly different characteristics. During the low-speed adjustment period (2000–2010), the coupling coordination degree rose from effective zero to 0.32, representing an annual average increase of 0.032 points. This initial phase reflected the healthcare system’s gradual recognition of and response to demographic aging, though the response lagged behind the pace of demographic change. The near-zero starting point in 2000 records a weak correspondence between the two composite indices rather than a measured shortfall of provision. Improvement over the decade was slow while healthcare reforms were under way, and we do not estimate what part either played. The coupling coordination degree improvement during this period was driven more by general healthcare system expansion than by targeted responses to aging. Notably, the coupling coordination degree increase (0.032 points annually) was proportionally lower than the aging coefficient increase (0.19 percentage points annually), so that over this period the aging index rose faster than the coordination index. This concerns the relative movement of two index series and not any gap between healthcare need and healthcare provision.

The rapid improvement period (2010–2023) witnessed a marked acceleration in coupling coordination degree enhancement, with the index rising from 0.32 to 1.00, representing an annual average increase of 0.052 points, against 0.032 points in the preceding decade. This phase transformation coincided with explicit policy recognition of population aging as a national strategic challenge, marked by the inclusion of aging response strategies in the 12th Five-Year Plan (2011–2015). The acceleration after 2015 was particularly pronounced, with the coupling coordination degree increasing from 0.54 to 1.00 in just seven years, a period over which integrated medical and long-term care policies for older adults were introduced and geriatric services established. Over the pandemic period (2020–2023) the index continued to rise. Concurrent healthcare investment and the deployment of digital health services are noted as context, their contribution to the observed movement was not estimated, and our indicators do not record which population groups used those services. The normalized score of 1.00 in 2023 is the pooled sample maximum and denotes a position relative to the other province-years in the panel, carrying no information about whether resources were adequate for any purpose.

Spatial patterns of the coupling coordination degree show geographic disparities that persist, and in some cases widen, alongside the improving national trend. Throughout the study period, coupling coordination degrees exhibited a dominant but non-monotonic east-high, west-low gradient that broadly paralleled economic development patterns. The gradient is a tendency rather than a rigid dichotomy: several western provinces combine high aging levels with relatively abundant healthcare resources and attain moderate-to-high coordination, notably Chongqing, whose aging coefficient reached 17.8% in 2023, Sichuan, at 17.1% and with a healthcare score of 0.64 already in 2000, and Shaanxi. Coordination therefore tracks the joint distribution of aging and resources rather than longitude as such, and the regional clusters used below are heuristic summaries in which intra-regional heterogeneity, particularly within the west, can exceed inter-regional differences. In 2000, eastern provinces already showed higher coupling coordination degrees despite the low national average, with Shandong (0.43) and Jiangsu (0.41) leading, while western provinces like Tibet (0.00) and Qinghai (0.05) showed low coordination between their limited aging and scarce healthcare resources. This initial disparity is a difference between two composite indices. Whether it also tracks differences in administrative capacity is not something our indicators can separate.

By 2023, while absolute coupling coordination degrees had improved across all provinces, relative disparities persisted and even intensified in some dimensions. Eastern provinces achieved high coordination levels, with Shandong reaching 0.95 and Jiangsu 0.91, indicating relatively high coordination between their aged populations and healthcare resources. These provinces score high on both composite indices. Whether service delivery arrangements such as integrated medical and long-term care facilities for older adults, or geriatric hospitals, contribute to the pattern is not assessed here, since our indicators record the stock of general healthcare resources only. The Yangtze River Delta region as a whole achieved coupling coordination degree above 0.85, benefiting from economic integration that facilitated resource sharing and coordinated planning. Central provinces improved, with Henan at 0.90 and Hubei at 0.79. Investment in healthcare infrastructure and in regional medical centres took place over the same years and is noted as context rather than as a tested cause.

Western provinces continued to record low coupling coordination degrees. Tibet (0.12) and Qinghai (0.17) remained in the insufficient coordination zone despite improvements, with coupling coordination degrees only one-eighth of eastern provinces. Sparse healthcare resources, long distances between settlements and facilities, and limited fiscal capacity are the conditions usually cited for values of this kind, though none of them is measured here. The ratio between the highest and the lowest provincial coupling coordination degree in 2023 was approximately 7.9. We did not identify comparably constructed subnational series for other countries and therefore make no international comparison.

A few provinces depart from the general gradient. Guangdong combines a high resource score with a comparatively low ageing score and records a moderate coupling coordination degree of 0.73, its migration history being offered as a reading rather than as a tested explanation. Heilongjiang shows a declining coordination value alongside a rising resource score, the ageing index having risen faster than the resource index over the same years. Beijing and Shanghai, with their relatively abundant healthcare resources, showed surprisingly moderate coupling coordination degrees (0.65 and 0.68 respectively) when adjusted for their severe aging, indicating that even resource-rich regions faced challenges in achieving high coordination.

## 5 Discussion

### 5.1 Spatiotemporal heterogeneity and emerging patterns in China’s aging healthcare dynamics

This section anchors each theoretical claim to a specific empirical result reported above. The epidemiological-transition discussion draws on the coexistence of multiple aging stages across provinces documented in Section 4.2; the inverse-care-law argument draws on the weak aging–resource association and the persistently low western coupling coordination degrees reported in Section 4.5; and health-equity theory draws on the concentration of resources in higher-aging developed regions shown in Sections 4.4 and 4.5. Read against epidemiological transition theory (2), our results indicate that China’s compressed demographic transition is associated with spatiotemporal patterns not anticipated by classical theory. While Omran’s model assumes gradual, broadly linear progression through demographic stages (2), we document simultaneous existence of multiple transition phases across provinces, a pattern we describe as demographic multiplicity. This bears on the generality of transition models derived from Western experience, and it sits alongside recent critiques of linear demographic accounts in Asian settings.

The inverse care law can be read into aging societies on the same evidence (19). While Hart’s original formulation focused on socioeconomic disparities, our results indicate that in China’s context, the inverse relationship between healthcare availability and population need operates through age-geographic intersections (29).

A further finding concerns the pronounced spatial heterogeneity in China’s aging process, which has narrowed only marginally over the study period. The spatial heterogeneity identified in our study (a coefficient of variation of the provincial aging coefficients of 0.38 in 2023, down from 0.41 in 2000) is large in absolute terms. We did not identify comparably constructed sub-national estimates for other rapidly aging countries and therefore draw no cross-national comparison. The pattern is consistent with the expectation that late-developing countries experience greater subnational demographic disparities as a result of uneven modernization.

However, our findings diverge from their prediction of eventual convergence; instead, we observe persistent and even widening gaps in specific regions, particularly the northeast-southwest axis. The emergence of what we term “multiple aging Chinas” extends beyond simple regional variations documented in prior work. The northeastern provinces have evolved into a “super-aged cluster” with aging coefficients exceeding 17.5%, effectively experiencing demographic conditions projected for China nationally in 2035 (17). This regional acceleration is consistent with accounts of demographic change in post-industrial regions elsewhere; comparable sub-national figures for those regions were not compiled here, so no quantitative comparison is drawn.

The second major finding concerns the paradoxical nature of healthcare resource development. Contrary to the “resource concentration thesis” suggesting that healthcare resources naturally gravitate toward aging populations (41), our data show a weak correspondence between the two indices, a pattern in which the distribution of capacity appears to track economic signals more closely than demographic structure. A spatial mismatch between primary care supply and older populations has been reported at sub-national scale for South Korea (38), although that study uses a different measure and is not directly comparable with the correlation reported here. In our data the correlation between aging intensity and healthcare resource scores is 0.43, which is consistent with the view that market-oriented reforms weaken the demographic responsiveness of healthcare systems and with the inverse care law (19); the present design cannot test this interpretation.

The persistence of a 48-fold difference in normalized healthcare resource scores between provinces, despite targeted western development policies, is a large within-country disparity; comparably constructed sub-national indices for other large countries were not compiled, and no numerical comparison is made. This suggests that China’s particular combination of vast territory, diverse development levels, and market-socialist hybrid system is associated with persistent spatial inequalities that respond slowly to policy intervention (9).

The dispersion between provinces reported in Section 4.5 persists across the period, which raises the question of what sustains it. The present design locates where the correspondence between the two indices is weakest and does not establish why, so what follows is interpretive. We read it against institutional complementarity, whereby the effectiveness of one institutional arrangement depends on the presence of others (20), and each consideration below is a hypothesis for subsequent work rather than a finding of this study.

Provinces with higher levels of economic development also record higher healthcare resource scores in our data. This co-variation is descriptive; no regression model was fitted and no explained-variance statistic is claimed (Section 3.4). The relationship between economic development and the distribution of healthcare resources in China has been examined directly elsewhere (9). On similar reasoning, sustained net out-migration of younger residents would raise the aging coefficient of a sending province while thinning the pool from which its healthcare workforce is drawn, though we compiled no migration series and estimate no such relationship.

Two further considerations sit on the supply side. Low settlement density and long travel distances raise the unit cost of delivering services in the western provinces, and our indicators record the stock of resources rather than the cost of reaching them or the time it takes. Provincial governments also differ in fiscal capacity and in the timing with which ageing-related measures were adopted. Neither was measured here, and we make no claim about the length of any lag between demographic change and health system response.

The last consideration is market allocation. The inverse care law holds that the availability of care varies inversely with the need of the population served, and that this operates more completely where care is exposed to market forces (19). Our data are consistent with a weak association between ageing intensity and healthcare resource scores, but whether the mechanism the law proposes is at work lies beyond what this design can test.

These findings collectively suggest that China’s aging-healthcare dynamics represent not merely scaled-up versions of patterns observed elsewhere, but qualitatively distinct phenomena requiring novel theoretical frameworks and policy approaches.

### 5.2 From uniform policies to differentiated strategies: implications for healthcare system reform

The multifaceted findings of this study carry substantial implications for policy formulation that extend beyond China to inform global responses to population aging. Our evidence supports a shift from uniform national policies toward spatially differentiated, temporally dynamic, and systemically integrated frameworks. This shift aligns with recent calls for “precision public health,” but requires adaptation to demographic rather than epidemiological targeting (25).

The persistence of spatial mismatches despite substantial healthcare investments challenges fundamental assumptions in health policy literature. Traditional convergence theory predicts that resource gaps should narrow over time through catch-up effects. Our findings of persistent and even widening disparities suggest that in rapidly aging societies, demographic momentum can outpace convergence forces. This “demographic override effect” has been theorized, but has seldom been examined empirically at this scale. The implication is that passive convergence strategies relying on economic growth and gradual diffusion may be insufficient; active countervailing policies may be required to offset market-driven concentration (34) (Table 5).

**Table 5:** Derivation of the policy directions from the empirical findings.

Empirical finding (source section)	Province group	Observed 2023 coordination	Derived policy direction
Lowest coordination values in the panel, with both subsystem scores low (Section 4.5)	Tibet, Qinghai	D = 0.12 and 0.17	Capacity supplementation is prior to optimization: the binding constraint is the absolute level of both subsystems, not their balance, so instruments that redistribute existing capacity cannot apply.
Moderate coordination with aging rising faster than capacity (Sections 4.1, 4.5)	Central provinces	Intermediate classes	Capacity building targeted at the service composition implied by the aging profile, since the gap is one of growth rate rather than of level.
Highest coordination values with capacity concentrated in agglomerations (Sections 4.4, 4.5)	Shandong, Jiangsu and comparable eastern provinces	D = 0.95 and 0.91	Redistribution within the province rather than further aggregate expansion, since the aggregate constraint is not binding while the internal distribution is.
High aging with capacity growth below the national trajectory (Sections 4.2, 4.5)	Northeastern provinces	Declining relative position	Instruments addressing workforce retention and out-migration, since the divergence originates on the demographic side rather than in investment.

No lag between demographic change and health system response was estimated in this study, and no such quantity is identified by the present design. Institutional accounts of path dependence and increasing returns (11, 12) nevertheless suggest why adjustment of the healthcare stock should be expected to be slow. Planning cycles are tied to the five-year plan and leave limited scope for mid-cycle adjustment, the training of specialist staff takes years, and infrastructure investment carries a long horizon. These are offered as hypotheses that a design with an explicit temporal identification strategy could test.

For regions with critically low coupling coordination degrees, our analysis suggests that conventional healthcare delivery models, premised on facility-based care and professional specialization, may be poorly suited. This finding resonates with “reverse innovation” concepts, where resource-constrained settings pioneer novel approaches later adopted by resource-rich areas. The challenging conditions in Tibet and Qinghai (coupling coordination degrees of 0.12 and 0.17) suggest a need to reconsider the delivery of care for older adults, potentially through (1) drone-based medication delivery adapted to harsh terrain; (2) AI-assisted diagnosis enabling non-specialists to manage complex geriatric conditions; and (3) community health worker models in which local residents receive training in geriatric care. These are illustrative options drawn from the wider policy literature rather than findings of the present study.

The concentration of resources in eastern urban centers, while creating local abundance, may generate inefficiencies of the kind economic theory anticipates. The concentration of resource scores in the eastern provinces documented in Section 4.4 is consistent with accounts of agglomeration under increasing returns in economic geography, in which activity concentrates in core regions (13); the criteria against which the resulting distributions may be judged are reviewed in the health economics literature (5), and independent evidence of spatial clustering in Chinese healthcare resources over 2010 to 2021 is reported elsewhere (8). However, unlike commercial goods, healthcare’s merit good characteristics and human rights dimensions create ethical imperatives for spatial equity. Recent work on “healthcare geography” proposes hub-and-spoke models that preserve agglomeration benefits while extending access. Our results give some reason to think such models would need adjustment for care of older adults, for whom travel burdens weigh more heavily (3).

Where previous studies examined aging and healthcare resources separately or at single time points, our 24-year panel analysis with coupling coordination models provides a systematic quantification of dynamic coordination relationships. The coupling coordination degree model, adapted from ecological economics, captures both the development of each system and the balance between them, which correlation-based approaches do not.

The identification of multiple aging Chinas also goes beyond the regional variation documented in prior work, since the provinces differ in the shape of their trajectories and not only in the level of the indices. This conceptualization aligns with recent “varieties of aging” frameworks in comparative gerontology, though what we add is granularity rather than a revision of the framing itself.

The Four-Dimensional Collaborative Development Framework, in turn, cuts across the binary urban–rural and east–west framings that dominate current policy discourse. It sets out differentiated planning directions by coordination type, which is a response to the limits of uniform prescriptions rather than a substitute for evaluating them.

The policy implications extend beyond China to inform global responses to population aging. As the WHO World report on ageing and health notes, populations are ageing rapidly worldwide and a coherent public health response spanning multiple sectors is required (1). Our findings suggest that adaptation is likely to require: (1) predictive rather than reactive planning systems that anticipate demographic changes by 5–10 years; (2) explicit mechanisms to counteract market-driven resource concentration; (3) innovation in service delivery models for low-density aging regions; and (4) international cooperation to share innovations across similar demographic-geographic contexts. The Chinese experience, despite its distinctive characteristics, offers lessons for nations confronting the dual challenges of population aging and spatial inequality (17).

### 5.3 Mechanisms of spatiotemporal heterogeneity: theoretical insights and international dialogue

Three processes are commonly invoked to account for spatiotemporal heterogeneity of the kind documented here, and we consider each against our results. The account that follows is interpretive: the study estimates no mechanism, and each process is offered as a hypothesis consistent with the observed patterns rather than as a result established here.

#### 5.3.1 Path-dependent demographic divergence

Provincial aging trajectories may carry a path dependency arising from how birth control policy was implemented and from migration. Provinces where implementation was stricter, such as Jiangsu and Sichuan, now face faster aging as smaller cohorts reach working age, while Tibet and Xinjiang retain younger structures. The onset of aging is therefore spread over a considerable interval across provinces, though we did not date that onset province by province and give no figure for the spread.

The “migration-selection mechanism” intensifies these disparities. Sustained net out-migration from the northeastern provinces would work in both directions at once, raising the aging coefficient while thinning the healthcare workforce, since it is the younger and better-educated residents who leave. We compiled no migration series, so the size of that outflow is not reported here and no relationship is estimated.

#### 5.3.2 Economic geography and cumulative causation

Healthcare resource concentration may follow the reinforcing patterns described for economic agglomeration. Existing capacity plausibly attracts further investment, which is what increasing returns would predict (13). We did not estimate any such relationship, and doing so would require a specification this study does not fit.

This concentration is amplified by patient mobility. Movement of patients toward the major centres is widely reported and would create incentives for continued concentration, though we compiled no data on patient flows and make no quantitative claim. This may indicate that domestic patient movement within a large country generates agglomeration forces of its own; comparable figures for international medical travel were not compiled and no comparison is made.

#### 5.3.3 Institutional fragmentation and geographic constraints

China’s healthcare heterogeneity is sustained by institutional and geographic factors that create persistent barriers to convergence. The “fiscal federalism paradox” operates through misaligned incentives: provincial governments bear healthcare funding responsibility while differing substantially in fiscal capacity, and inter-governmental transfers narrow, without closing, the resulting gaps.

Geographic constraints impose limits on service delivery that technology may not fully overcome. Distance decay in healthcare utilization is steep in Tibet, and topography and climate create seasonal isolation for a substantial share of Xinjiang’s rural older population. Comparable figures for other high-latitude or high-altitude regions were not compiled and no comparison is drawn.

These mechanisms interact synergistically: path-dependent aging creates differential demands, economic agglomeration concentrates resources away from needs, while institutional and geographic barriers constrain corrective redistribution. Taken together these processes would, if operative, work against spatial convergence. Whether they do so is not established by the present analysis, and testing them would be a useful step toward designing interventions that work with rather than against them (33).

#### 5.3.4 International comparison

The pattern documented here can be placed against the experience of the other rapidly aging systems of East Asia and of Europe, and the comparison locates what is distinctive about the Chinese case. Japan crossed the 14% threshold in 1994 and reached 21% by 2007, and its response was to establish a dedicated long-term care insurance program in 2000, financed separately from medical insurance and organized around community-based integrated care, so that the expansion of capacity for older adults occurred through a channel constructed for that purpose (44, 45). South Korea introduced its long-term care insurance in 2008, when older adults were still about a tenth of the population, which made the response anticipatory rather than reactive (46). In both cases the institutional response was a financing instrument specific to long-term care, and in both cases it preceded the steepest part of the demographic curve. The Chinese trajectory documented in this study differs in mechanism. The improvement in the coupling coordination degree over 2010–2023 is driven by the expansion of general healthcare capacity, since general capacity is what our indicators record and since the long-term care insurance pilots begun in 2016 remain partial in provincial coverage. This has two consequences that the international comparison brings out. The coordination gains reported here may be less durable than they appear, because general capacity expansion is not targeted at the service types that the composition of aging-related need calls for. The interprovincial dispersion documented here, in turn, has no clear counterpart in Japan or Korea, whose smaller territory and nationally financed schemes limit subnational divergence; the comparison therefore suggests that the spatial dimension is not incidental to the Chinese case but constitutive of it, and that a nationally uniform financing instrument of the Japanese or Korean type would encounter, in China, a distribution of starting conditions that those systems did not face.

### 5.4 Methodological constraints and data limitations in provincial-level analysis

While this study provides comprehensive insights into the spatiotemporal dynamics of aging and healthcare resources in China, several limitations warrant careful consideration when interpreting findings and deriving policy implications.

The reliance on provincial-level administrative data, while necessary for data completeness and comparability across the 24-year study period, masks substantial intra-provincial variations that may be as significant as inter-provincial differences. China’s provinces, particularly in the west, encompass territories larger than many countries, with internal diversity in geographic conditions, economic development, and demographic structures that provincial averages do not capture. For instance, Xinjiang’s provincial aging coefficient obscures marked variations between the relatively young southern prefectures with high proportions of ethnic minorities and the aging northern regions dominated by Han populations. Similarly, provincial healthcare resource scores fail to capture the concentration of resources in provincial capitals versus the sparse distribution in peripheral areas. In several western provinces the higher-level medical resources are concentrated in the provincial capital while extensive mountainous areas are served far more thinly. We did not compile sub-provincial data and give no figure for that concentration. This spatial aggregation problem is particularly acute for policy formulation, as provincial-level policies based on average conditions may be inappropriate for the diverse sub-provincial realities.

A constraint on inference follows from the unit of observation and should be stated in the terms in which it will be encountered by readers. Every quantity in this study is a provincial aggregate, and the coupling coordination degree is a property of a province-year, not of a person. It follows that a high coordination value is not evidence that any individual older adult in that province used healthcare services, had their healthcare needs met, or experienced better health outcomes, and a low value is not evidence of the contrary for any individual. Inferring individual-level relationships from these aggregates would constitute an ecological fallacy. The provinces are also large and internally heterogeneous, so that a provincial value can conceal opposing movements within the province, as the concentration of capacity in provincial capitals illustrates. The findings should therefore be read as a description of the regional-level correspondence between demographic structure and healthcare capacity, which is informative for the planning of that capacity, and not as evidence about the healthcare experience of older adults, which requires individual-level data of a kind this design does not use.

Our coupling coordination degree model, while providing useful insights into aging-healthcare coordination, embodies several simplifying assumptions that may not fully reflect the complex realities of healthcare demand and supply. The assumption of equal importance (α = β = 0.5) between aging intensity and healthcare resources implies a linear relationship between demographic aging and healthcare needs, yet evidence suggests that healthcare utilization increases exponentially in advanced age groups. The model also assumes substitutability between different types of healthcare resources, when in reality, older adults require specific services that general healthcare resources may not adequately replace. Furthermore, the model treats all older populations as homogeneous in their healthcare needs, overlooking significant variations based on factors such as urban versus rural residence, socioeconomic status, family support structures, and cultural attitudes toward healthcare utilization. These simplifications, while necessary for tractable analysis, mean that high coupling coordination degrees may coexist with substantial unmet needs for specific subgroups of older adults or particular types of services.

Data constraints imposed several additional limitations on our analysis. The exclusion of private healthcare resources, necessitated by inconsistent data collection and reporting standards for private facilities, potentially leads to systematic underestimation of total healthcare resources, particularly in economically developed regions where private healthcare has expanded rapidly. Private hospitals had come to account for roughly two thirds of all hospitals in China by 2020 (47), though their contribution to care for older adults is unclear, since they concentrate in profitable specialties rather than in geriatric services. The study period ending in 2023 also prevents analysis of post-pandemic adjustments in healthcare resources and the potential long-term impacts of COVID-19 on the healthcare needs of older adults. The reliance on quantity-based indicators for healthcare resources, while supporting data availability and comparability, does not capture quality dimensions that may be equally or more important for the effectiveness of care for older adults. A province may show abundant hospital beds but lack geriatric expertise, or possess numerous primary care facilities staffed by providers without training in the care of older adults (27).

Methodological limitations also affect our findings’ interpretation. The use of principal component analysis, while effective for dimension reduction, creates composite indices whose specific meaning can be opaque and whose temporal comparability may be affected by changing variable relationships over time. The entropy weight method, despite its objectivity in weight assignment, may overemphasize variables with high variation that may not necessarily be most important for the care of older adults (31). The spatial analysis treats provinces as independent units, yet significant spillover effects exist through patient mobility, resource sharing, and policy learning that our methods do not fully capture. Provinces do not exist in isolation but form parts of regional healthcare systems, particularly in economically integrated regions like the Yangtze River Delta, where administrative boundaries poorly reflect functional healthcare service areas.

### 5.5 Advancing the research frontier: multi-scale analysis and technological innovation

The most immediate research priority involves disaggregating analysis to sub-provincial levels, particularly county and prefecture units that better reflect functional healthcare service areas and local demographic realities. County-level analysis would reveal the true extent of aging-healthcare mismatches obscured by provincial averaging and enable identification of local best practices in achieving coupling coordination despite resource constraints. Such analysis requires overcoming substantial data challenges, as county-level health statistics remain incomplete and inconsistently reported across China. Innovative approaches combining administrative data, survey information, and big data sources could construct more granular pictures of aging patterns and healthcare utilization. Research should particularly focus on rural counties experiencing rapid aging due to youth out-migration, where conventional healthcare delivery models face the greatest challenges.

Beyond spatial disaggregation, temporal dynamics warrant sophisticated investigation through predictive modeling that integrates demographic projections with healthcare system evolution. Current reactive approaches to healthcare planning could be transformed through machine learning models that predict future aging-healthcare mismatches based on demographic momentum, policy scenarios, and socioeconomic trajectories. Such models should incorporate uncertainty quantification to provide ranges rather than point estimates, enabling robust planning under various scenarios. Particular attention should focus on identifying “tipping points” where gradual demographic changes may trigger rapid healthcare system stress, enabling preemptive interventions. Agent-based models could simulate how individual-level behaviors aggregate to create system-level aging-healthcare dynamics (36).

The quality dimensions of healthcare resources, largely absent from current quantity-focused analyses, require systematic investigation through novel measurement approaches. Research should develop and validate quality indicators specific to the care of older adults, such as geriatric competency indices for healthcare providers, age-friendliness scores for healthcare facilities, and integration measures for medical-social service coordination. Mixed-methods approaches combining quantitative metrics with qualitative assessments of patient experiences could provide holistic pictures of the quality of care for older adults. Comparative studies across provinces with similar resource quantities but different care models could identify quality-enhancing practices transferable to other contexts. The development of comprehensive databases on the quality of care for older adults, possibly through mandatory reporting systems, would enable continuous quality monitoring and improvement (7).

Digital health technologies emerge as a critical research frontier given their potential to transcend geographic barriers constraining traditional healthcare delivery. Research should investigate suitable designs for digital health systems for older adults that address usability challenges, trust concerns, and infrastructure limitations in remote areas. Randomized controlled trials of digital health interventions specifically targeting older adults in resource-scarce areas could establish evidence bases for scaled implementation. The integration of artificial intelligence in the care of older adults, from diagnostic support to care coordination, requires careful study of both effectiveness and ethical implications. Research should also examine digital divides among older adults and strategies for ensuring equitable access to technology-enabled care.

The intersection of family dynamics and formal healthcare systems in the provision of care for older adults deserves expanded investigation given China’s traditional reliance on family support and ongoing changes in family structures. Research should quantify the substitution and complementarity relationships between family care and formal healthcare services across different cultural contexts within China. The impacts of changing family structures, including declining fertility, increased geographic mobility, and evolving gender roles, on healthcare demand among older adults require longitudinal study. Policy experiments with family support programs, such as compensation for family caregivers or training programs for family members, could inform appropriate balances between family and formal care systems.

Finally, China’s experience as one of the largest and most rapidly aging societies positions it as an important case for international comparative research. Studies comparing China’s aging-healthcare dynamics with those of other Asian societies undergoing rapid demographic transitions could identify culturally adapted solutions to common challenges. Comparative analysis with developed countries that aged more gradually could distinguish which challenges are inherent to population aging versus specific to rapid transitions. China’s diverse provincial experiences also offer natural experiments for policy learning, where different approaches to similar demographic challenges enable causal inference about policy effectiveness. The development of internationally comparable indicators for aging-healthcare coordination would facilitate global learning and adaptation of successful interventions across contexts.

The empirical findings of this study have important implications for theoretical development in related fields. The results support and extend the application of Andersen’s Behavioral Model of Health Services Use at the macro level. We found that inter-provincial differences in aging levels are associated with differences in healthcare resource demand patterns, supporting the role of population structure as “predisposing characteristics” in healthcare service demand, while revealing the applicability of this theory in regional comparative research. The study also speaks to health equity theory. The identified coupling coordination degree differences between eastern and western regions indicate that China’s healthcare resource allocation exhibits a “reverse allocation” pattern, more developed regions with higher aging levels possess more abundant healthcare resources, while less developed regions experiencing accelerated aging face resource shortages. This finding challenges traditional “resource equalization” policy thinking and supports needs-based differentiated allocation strategies. It further develops the application of coupling coordination theory in social system research. We found that there is a discernible pattern of evolution from “discoordination” to “coordination” between population aging and healthcare resource allocation, and this evolutionary process is jointly influenced by economic development levels, policy interventions, and spatial spillover effects. This provides empirical support for understanding the co-evolution mechanism among social subsystems and enriches the application scenarios of coupling coordination theory.

### 5.6 From coupling coordination degree to coupling coordination efficiency: conceptual boundaries and policy implications

One conceptual boundary is worth stating explicitly, since it bears on how the contributions and limitations of this study should be read. The coupling coordination degree used here summarizes the statistical correspondence between two composite indices. It does not measure whether supply-side capacity corresponds to demand, and the discussion in this section is framed accordingly. However, high coupling coordination degrees should not be interpreted as evidence of efficient resource utilization or genuine satisfaction of the health needs of older adults.

For instance, a province achieving D > 0.60 may have done so through construction of high-end healthcare facilities that meet aggregate quantitative criteria but fail to address the actual needs of older adults with ordinary incomes. The eastern provinces’ high coupling coordination degrees may reflect resource abundance that exceeds actual utilization, while potentially masking inequities in service accessibility and affordability. Similarly, rapid resource expansion may involve substantial costs or opportunity costs not captured by our supply-side indicators. A region might rapidly improve its indicators by constructing numerous high-end nursing homes, yet this might not meet the actual needs of the majority of older adults with modest incomes.

This conceptual boundary has important policy implications. Our “Four-Dimensional Collaborative Development Framework” should be understood as addressing quantitative structural alignment rather than prescribing efficiency improvements. Policymakers implementing these recommendations should complement quantity-focused strategies with quality assurance mechanisms, including: (1) monitoring health outcome indicators such as mortality among older adults, chronic disease management effectiveness, and patient-reported outcomes; (2) assessing resource utilization efficiency through bed occupancy rates, outpatient-to-inpatient ratios, and cost-per-service metrics; (3) evaluating equity dimensions including out-of-pocket expenditure burdens, geographic accessibility adjusted for mobility limitations, and service utilization rates across income groups; and (4) conducting regular needs assessments to examine whether resource types match the actual preferences of older adults rather than assumed demand. The fundamental objective should remain improving the intrinsic quality and fairness of the healthcare service system, rather than merely pursuing “coordination” in quantitative indicators.

Future research should integrate efficiency and quality indicators as these become available in China’s evolving health statistics system. Assessments of the first decade of the 2009 reform report that gains in coverage were not matched by comparable gains in efficiency and quality (48), which is precisely the dimension the coupling coordination degree does not capture. The establishment of the National Healthcare Security Administration (2018) and expansion of long-term care insurance pilots have generated new data streams that may enable efficiency assessments in subsequent studies. Comparative studies examining provinces with similar coupling coordination degrees but different efficiency outcomes could illuminate the factors that translate structural alignment into genuine health improvements for older adults.

Several limitations should be recorded. Data availability constrained the healthcare resource indicators to comprehensive indicators for the general population and do not include indicators specific to older adults such as geriatric beds, long-term care facilities, and geriatric medical personnel. Although we have justified the validity of comprehensive indicators as proxy variables, this limitation may affect the precision of coupling coordination degree assessment to some extent. Future research should incorporate more targeted specialized indicators as China’s statistics on healthcare resources for older adults improve. The analysis is also set at provincial level, which may mask internal disparities between urban and rural areas and among different prefectural cities within provinces. It rests on supply-side indicators and does not incorporate demand-side indicators such as actual healthcare service utilization and health outcomes among older adults. The analysis is descriptive rather than inferential: no panel estimator with fixed or random effects, no regression model and no spatial econometric specification was fitted, so the differences reported between provinces and periods are observed differences in the sample rather than effects estimated against a null hypothesis, and no causal claim is made. Related to this, global and local spatial autocorrelation statistics such as Moran’s I and local indicators of spatial association (21) were not computed, so the clustering described in Sections 4.2, 4.4 and 4.5 is a visual and classification-based description rather than a statistically tested pattern; estimating spatial autocorrelation and spatial spillovers, and complementing the index with panel models that exploit the 24-year structure, are the two most immediate methodological extensions of this work. Finally, the framework captures structural correspondence but not efficiency or quality. High coupling coordination degrees do not inherently indicate that resources are utilized effectively, that the health needs of older adults are genuinely met, or that alignment has been achieved at reasonable cost. This supply-side focus, while necessary given data constraints, means that our policy discussion addresses quantitative resource capacity rather than service delivery quality or efficiency. Future research incorporating outcome-based indicators would contribute to a fuller understanding of aging-healthcare system performance. These limitations provide directions for improvement in subsequent research.

## 6 Conclusion

This study assesses the spatiotemporal coordination between two provincial composite indices, one of population aging characteristics and one of healthcare resource capacity, for 31 Chinese provincial-level regions over 2000–2023. What is assessed is the correspondence between two statistical constructs. The study does not estimate causal effects, does not measure the matching of healthcare demand to healthcare supply, and does not observe the healthcare received by older adults. China’s aging coefficient doubled from 7.0 to 14.9% over the study period. This national aggregate masks substantial spatial heterogeneity: eastern provinces such as Liaoning reached a 20% share of the population aged 65 and above, a level projected nationally only for 2035, while western provinces maintain age structures resembling earlier decades, creating “multiple aging Chinas” within national borders. Healthcare resources expanded substantially in absolute terms, with hospital beds reaching 9.10 million and licensed physicians 4.28 million by 2023 and the normalized composite score rising from 0.02 to 0.91, yet the interprovincial distribution of the resource composite remained uneven, with the highest scores concentrated in the eastern provinces throughout the period. The national coordination index rose to the upper bound of the pooled min–max scale by 2023. This value denotes the maximum observed in the sample and not empirically perfect coordination; it would be attained by whichever province-year happened to rank highest, whatever the absolute level of provision. Beneath this national figure the interprovincial distribution remained wide, with Tibet at 0.12 and Qinghai at 0.17 against 0.95 in Shandong and 0.91 in Jiangsu, a ratio of approximately 7.9 between the extremes. The low values indicate that the two composite indices stand in a weak correspondence in those provinces; whether the healthcare needs of older adults there are met is a separate question that requires individual-level data.

These findings support a shift from uniform policies toward a Four-Dimensional Synergistic Development Framework. The four types form a rule-based typology defined on the two dimensions measured in this study: provinces are classified according to whether their 2023 aging coefficient and their 2023 healthcare resource score lie above or below the national median, yielding four categories of high aging with high resources, high aging with low resources, low aging with high resources, and low aging with low resources, each read against the coupling coordination degrees reported in Section 4.5. The typology follows from the joint distribution of aging and resources rather than from geography, which is why Sichuan, Chongqing and Shaanxi fall with the better-coordinated group despite their western location, while Tibet, Qinghai and parts of the northeast fall into the mismatched categories. Each type is linked to the mechanisms established in Section 5.3, namely path-dependent demographic divergence, cumulative causation in resource concentration, and institutional and geographic constraints, so that the strategies set out below follow from identified drivers. The strategies set out below follow the direction indicated by the typology; their specific instruments are illustrative options drawn from current policy practice rather than quantities derived from our data, and the targets, staffing levels and thresholds mentioned would need to be established by costing and implementation studies. Western regions require precision supplementation combining central fiscal commitments with adapted delivery models, such as extending geriatric department coverage in county hospitals, deploying certified specialists to township centres, and establishing telemedicine networks. Central provinces need capacity-building through regional medical centers for older adults in Zhengzhou, Wuhan, and Changsha, specializing in cardiovascular and orthopedic care, while pioneering “Medicare + Long-term Care Insurance” integrated payment systems. Eastern regions could optimize through satellite medical sub-centres and cross-provincial alliances to decompress congested facilities. Northeastern provinces call for transformative interventions, such as “Silver Medical Zones” as innovation demonstrations, targeted retention programs, and seasonal specialist rotations from southern provinces.

Putting these strategies into practice would require monitoring arrangements that anticipate rather than react. A national monitoring platform integrating multi-source data could flag emerging coordination gaps using tiered alerts anchored on the coupling coordination thresholds reported in Section 3.3.4, with the lowest tier triggering mobilization, the middle tier capacity building and the highest tier optimisation. Regional reserve centres and flexible specialist deployment could support emergency response, and technology-enabled solutions could help bridge geographic barriers through diagnostic equipment with minority language interfaces, AI-assisted village-level facilities, and periodic third-party quality evaluation. The number of such centres, the size of any specialist corps and the alert cut-offs are matters for implementation research; our data identify where the gaps are, not how large an intervention should be. Taken together, differentiated regional approaches, predictive monitoring and technological innovation offer pathways toward achieving quantitative structural alignment between aging populations and healthcare resources. However, achieving high coupling coordination degrees represents a necessary foundation rather than a final objective. Policymakers should complement quantity-focused interventions with quality assurance mechanisms so that structural alignment is more likely to translate into efficient resource utilization, genuine satisfaction of the diverse health needs of older adults, and equitable healthcare access. The measurement of coupling coordination efficiency, meaning how effectively aligned resources produce health outcomes, is one direction for future research and policy development. These lessons offer insights for nations confronting rapid aging amidst spatial inequalities.

The contribution of this study is threefold and can be stated precisely. It provides an assessment of aging–capacity coordination that covers all 31 provincial-level regions across the 24 years spanning China’s major healthcare reforms, using one indicator framework held constant throughout, so that the resulting series is comparable across provinces and over time. It documents that the improvement in the national coordination index over this period coexists with an interprovincial dispersion that has narrowed only marginally, the coefficient of variation of provincial aging coefficients moving from 0.41 to 0.38. And it converts these patterns into a rule-based typology of provinces defined on the two measured dimensions, which identifies where the correspondence between demographic structure and healthcare capacity is weakest. The implication is for planning rather than for evaluation: the results indicate which provinces should be prioritized when capacity is allocated under continuing demographic change, and they do not establish that any particular allocation would improve the care that older adults receive, which remains a question for evaluative and individual-level research.

## Data Availability

The datasets used in this study were compiled from published statistical sources identified in Section 3.1 and listed by indicator in Table 1. The compiled province-year panel and the parameter settings behind the composite indices are available from the corresponding author on request. Further inquiries can be directed to the corresponding author.
