# Supplementary material for: Spatiotemporal coupling coordination between population aging and healthcare resource capacity in China: a provincial-level analysis, 2000–2023
Source: Front Public Health. 2026 Sep 23;14:1835598. doi: 10.3389/fpubh.2026.1835598 (PMC13642027; doi:10.3389/fpubh.2026.1835598)
Supplement: Supplementary file 1 [file Table_1.DOCX]

Table S1. Result 1: Full sample vs. sample excluding Tibet and Xinjiang

| **Indicator** | **Full national sample (31 provinces, 713 valid obs)** | **Subsample excluding Tibet & Xinjiang (29 provinces, 682 valid obs)** | **Consistency judgment** |
| --- | --- | --- | --- |
| National aging coefficient growth trajectory (2000–2023) | 7.0% → 14.9%, two-stage “acceleration–stabilization” pattern; annual growth 0.19% (2000–2010), 0.46% (2010–2023) | 7.02% → 14.93%, identical two-stage growth rhythm; annual growth 0.188% (2000–2010), 0.462% (2010–2023) | Fully consistent, trend curve overlap >99% |
| Healthcare resource composite score trajectory (2000–2023) | 0.02 → 0.91, slow growth 2000–2010, rapid expansion after 2010 | 0.021 → 0.912, identical phase division and growth slope | Fully consistent, relative index change consistent |
| Core spatial pattern | Persistent east-high, west-low aging & healthcare resource gradient; Northeast super-aged cluster, western scarcity zone | Identical east–west spatial gradient; Northeast super-aged cluster, central mid-level, western low-value zone (only Tibet/Xinjiang removed) | Spatial classification pattern unchanged |
| National average coupling coordination degree (2023) | 1.00 (sample normalized maximum) | 0.997 (min–max normalized separately for 29 provinces) | Overall coordination level consistent |

Table S2. Result 2: Comparison of coupling coordination degree (D) under four imputation strategies

| **Imputation method** | **Pearson correlation r vs. baseline D** | **Spearman rank correlation ρ vs. baseline D** | **Max absolute deviation ΔD (single province-year)** | **Interpretation** |
| --- | --- | --- | --- | --- |
| Carry forward nearest observed value | 0.976 | 0.968 | 0.014 | Extremely high linear & rank consistency, negligible deviation |
| Cubic spline interpolation | 0.981 | 0.974 | 0.011 | Almost identical coordination values and provincial rankings |
| Province-specific linear extrapolation | 0.972 | 0.965 | 0.016 | Provincial coordination hierarchy fully preserved |

Table S3. Result 3: Classification stability test (imputed values replaced with upper/lower bound of adjacent actual observations)

| **Province** | **Index dimension** | **Classification under lower bound of adjacent data** | **Classification under upper bound of adjacent data** | **Classification result** |
| --- | --- | --- | --- | --- |
| Xizang (Tibet) | Population aging stage (aging coefficient) | Young society (<7%) | Lightly aging society (7–10%) | No cross-stage jump; always lowest aging tier in western region |
| Xizang (Tibet) | Coupling coordination level | Insufficient coordination (D<0.20) | Insufficient coordination (D<0.20) | Classification tier unchanged |
| Xinjiang | Population aging stage | Lightly aging society (7–10%) | Moderately aging society (10–14%) | Remains low-to-middle aging tier, no shift to high aging group |
| Xinjiang | Coupling coordination level | Insufficient coordination (D<0.20) | Coordination to be improved (0.20≤D<0.30) | Stays within the two lowest coordination tiers, no leap to medium/good coordination |

Table S4. Result 4: Impact of imputed cells on national normalized composite scores (2000–2003)

| **Year** | **Change range of national aging composite score after shifting imputed values to upper/lower bounds** | **Change range of national healthcare resource composite score after shifting imputed values to upper/lower bounds** | **Maximum absolute index fluctuation** |
| --- | --- | --- | --- |
| 2000 | ±0.004 | ±0.003 | 0.004 |
| 2001 | ±0.005 | ±0.004 | 0.005 |
| 2002 | ±0.004 | ±0.003 | 0.004 |
| 2003 | ±0.006 | ±0.005 | 0.006 |
